# AhR-dependent ferroptosis as a therapeutic opportunity to counteract BRAFi-resistance in melanoma

**DOI:** 10.1038/s41420-026-03057-3

**Published:** 2026-03-23

**Authors:** Cyrille Berra, Héloïse M. Leclair, Anthony Sebillot, Diane Schausi, Justine Guillo, Maria Francesca Baietti, Eleonora Leucci, Marie-Dominique Galibert, Sébastien Corre

**Affiliations:** 1 Univ Rennes, CNRS, INSERM, IGDR (Institut de Génétique et Développement de Rennes) – UMR6290, ERL U1305, University of Rennes, Rennes, France; 2 Department of Molecular Genetics and Genomics, Hospital University of Rennes (CHU Rennes), Rennes, France; 3 CNRS, Inserm, Biosit UAR 3480 US_S 018, France-BioImaging (ANR-10-INBS-0005 FBI BIOGEN), Core Facility H2P2, University of Rennes, Rennes, France; 4 Department of Oncology, LKI, Trace PDX Platform, KU Leuven, Leuven, Belgium; 5 Laboratory for RNA Cancer Biology, Department of Oncology, LKI, KU Leuven, Leuven, Belgium

**Keywords:** Melanoma, Cancer metabolism

## Abstract

Drug resistance limits the achievement of persistent cures for the treatment of melanoma, despite the efficacy of targeted therapies. This study explored how transcriptional regulation governs metabolic adaptations that underlie resistance. Our analysis of the metabolic profiles revealed a distinct shift in resistant melanoma cells—from glycolytic metabolism in BRAFi-sensitive cells to oxidative phosphorylation (OXPHOS) dependence. This transition was accompanied by a reprogramming of transcriptional networks, marked by the downregulation of MITF transcription factor and a pronounced upregulation and activation of the Aryl hydrocarbon Receptor (AhR). AhR emerged as a key regulator of this resistant phenotype, contributing to the metabolic switch that enhances mitochondrial function, elevates reactive oxygen species (ROS) production, and drives lipid peroxidation. This reprogramming sensitizes resistant cells to ferroptosis, a regulated cell death driven by iron-dependent lipid peroxidation. Importantly, pharmacological activation or stabilization of AhR exacerbated this susceptibility, while its inhibition mitigated ferroptotic responses—highlighting AhR not only as a mediator of resistance-associated metabolic rewiring but also as a potential therapeutic target. Collectively, these findings position AhR as a central node linking metabolic plasticity to ferroptosis vulnerability, offering a novel axis for therapeutic intervention in drug-resistant melanoma.

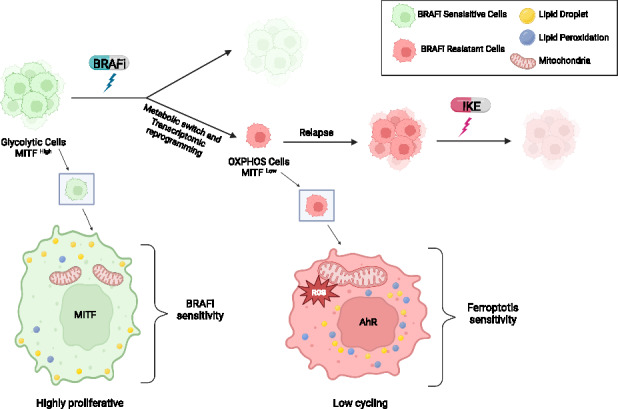

## Introduction

Metabolic rewiring is a hallmark of malignant transformation across diverse cancer types [1–3]. In the tumor microenvironment, cancer cells face oxygen and nutrient deprivation, prompting metabolic adaptations for survival. The increased demand for energy to support rapid proliferation necessitates significant changes in the carbon metabolism. Invasive tumor cells, which aim to escape the constraints of the primary tumor and invade distant sites, require metabolic plasticity. This flexibility serves two purposes: first, to sustain the energy-intensive migratory process [4] and second, to endure the nutrient-depleted environments they may encounter beyond the primary site. Many types of cancer, including melanoma, rely heavily on metabolic adaptations to fuel their growth and confer resistance to therapeutic treatment.

Melanoma is one of the most prevalent cancers in western countries, and its incidence continues to rise [5]. The complexity of this aggressive cancer relies on the presence of different cell subpopulations within the same tumor, each of which presents distinct phenotypes. Glucose, glutamine [6], and fatty acids [7, 8] metabolic reprogramming, characteristic of cellular transformation, is pivotal for melanoma cells to adopt the phenotypic states necessary for environmental adaptation.

In 2011, the treatment of advanced melanoma underwent significant transformations owing to the use of immunotherapy [9] and targeted therapies [10–12]. Selective inhibitors targeting the V600E mutated form of the BRAF kinase have extended the survival of patients harboring this mutation [11, 13, 14]. However, the emergence of resistance necessitates the combination of BRAF and MEK inhibitors. This combination constitutes the current standard of care [10, 12, 15]. Despite offering more prolonged disease control compared to BRAF inhibitors alone, dual therapy is hindered by the emergence of drug resistance [16]. Growing evidence suggests that melanoma cells resistant to a double BRAF/MEK blockade exhibit distinct metabolic profiles characterized by specific transcriptional programs. These cells possess the capability to transiently alter their phenotype under treatment to assume an invasive and treatment-resistant state, which is characterized by slow-cycling cells and elevated oxidative phosphorylation (OXPHOS) metabolism [17].

Exposure of xenografted mice with tumors derived from BRAF mutant melanoma patients (PDX) to MAPK inhibitors has revealed significant complexity: up to four distinct subpopulations of drug-tolerant cells coexist within the same post-therapy minimal residual disease (MRD), arising from non-mutational adaptive events. These states exhibit variable levels of the melanocyte-specific b-HLH-Zip protein, Microphthalmia-associated Transcription Factor (MITF). Its expression and activity range from high (pigmented state), to intermediate (starved state) and low (invasive and neural crest stem cell states) [18–20]. MITF is a crucial regulator of phenotypic identity and fate in melanoma cells, and is capable of driving both differentiation and proliferation [21]. Notably, reduced MITF levels are linked to invasion, enhanced tumor-initiating capacity [22, 23], and emergence of drug-resistant, slow-cycling phenotypes [18, 20]. Importantly, nutritional and metabolic environmental signals can reduce MITF expression at both transcriptional and translational levels [8, 24–26]. Conversely, MITF significantly shapes the metabolic landscape by providing both the energy required for increased cellular replication and the building blocks for synthesizing vital macromolecules such as membrane components and DNA. MITF controls the tricarboxylic acid (TCA) cycle by directly modulating the activity of succinate dehydrogenase (SDHB), thereby influencing succinate levels [27]. MITF governs the expression of PGC1α, orchestrating OXPHOS [28], and serves as a pivotal inducer of the lipogenic enzyme stearoyl-CoA desaturase (SCD), which regulates the degree of fatty acid (FA) saturation, crucially determining the balance between intracellular long-chain saturated fatty acids (SFAs) and monounsaturated fatty acids (MUFAs). Together, these findings support the proliferation of MITF^high^ melanoma cells [8].

Research on the adaptability of melanoma cells has mainly focused on deciphering the role of MITF. However, our recent investigations have revealed that the Aryl hydrocarbon receptor (AhR), a ligand-dependent transcription factor belonging to the b-HLH-PAS family, significantly contributes to the progression of MITF^low^ cells. We uncovered the ability of AhR, once activated, to promote resistance to targeted therapy [29] and further elucidated its pivotal role in orchestrating melanoma plasticity, maintaining a dedifferentiated and invasive phenotype [30]. Comprehensive metabolomic analysis in Ahr−/− and Ahr+/+ mice have revealed significant alterations in circulating levels of hundreds of metabolites upon Ahr loss [31] and since AhR has been shown to play a critical role as a regulator of the antioxidant response within cells [32], we sought to uncover the role of AhR in metabolic rewiring and melanoma resistant cells to identify novel therapeutic vulnerabilities.

We further investigated melanoma-resistant cells transitioning towards an OXPHOS metabolism, accompanied by increased mitochondrial activity and the accumulation of reactive oxygen species (ROS). Additionally, we demonstrated that BRAFi-resistant cells activate various metabolic pathways, including glutathione metabolism and polyunsaturated lipid peroxidation, leading to the predominant induction of ferroptosis. Consequently, we identified a therapeutic vulnerability in resistant BRAFi cells, which appears to depend on the expression and activity levels of the AhR transcription factor. Treatment with diverse ferroptosis inducers holds promise for addressing resistant melanoma cells and open new avenues for patient care.

## Results

### BRAFi resistance is closely linked to metabolic alterations

**Fig. 1:**
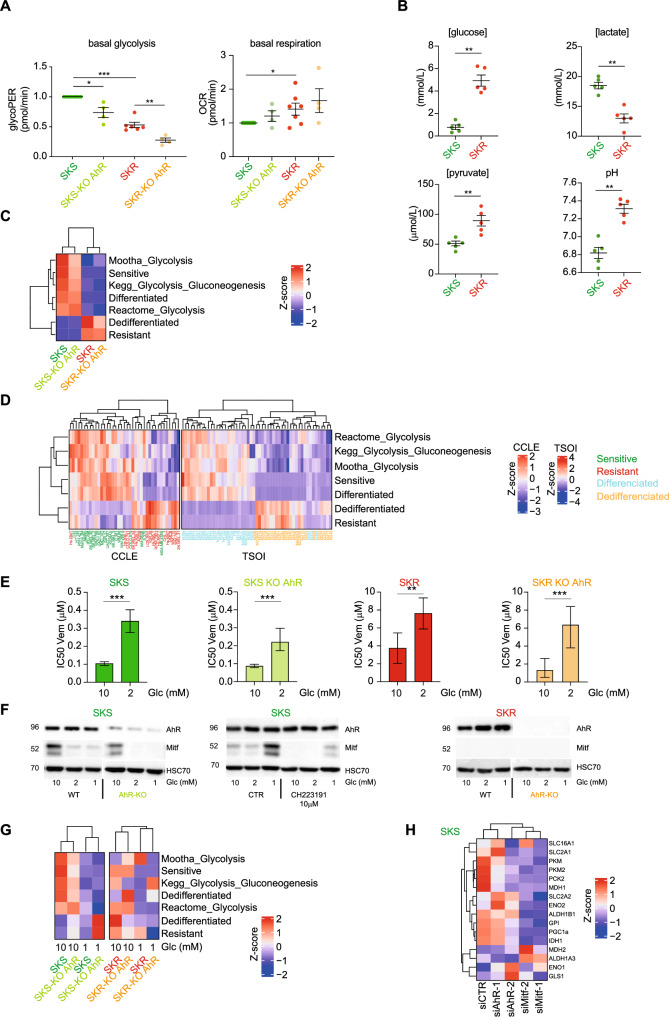
Metabolic change in BRAFi resistant cells. **A** Seahorse analysis of sensitive (SKS) and resistant (SKR) SKMel28 cells invalidated or not for AhR transcription factor (KO) by CRISPR/Cas9 to determine the glycolytic proton efflux rate (glycoPER) and oxygen consumption rate (OCR) in cells (mean + /sem; *n* = 4–7; unpaired Mann-Whitney *t*-test; *p* < 0.05*; *p* < 0.01**; *p* < 0.001***). **B** Measure of pH, glucose, lactate, and pyruvate concentration in supernatant from cell culture medium for SKS or SKR after 24 h (mean + /sem; *n* = 5; unpaired Mann-Whitney *t*-test; *p* < 0.05*; *p* < 0.01**; *p* < 0.001***). **C** Expression heatmap for various gene signatures (from Appendix Table S1 [30] and GSEA from Table S1) in SK28 BRAFi-sensitive or resistant cell lines KO or not for AhR from RNAseq data (GSE166617). Genes and clusters with similar expression profiles across the cohort are placed close to each other in the grid. The scale corresponds to the Z scores. **D** Expression heatmap for various gene signatures in BRAFi-sensitive or resistant melanoma cell lines from the Cancer Cell Line Encyclopedia (CCLE) [33] and differentiated or dedifferentiated melanoma cell lines from the Graeber data sets [19]. Genes and clusters with similar expression profiles across the cohort are placed close to each other in the grid. The scale corresponds to the Z scores. **E** BRAFi sensitivity was established in SKMel28 cells grown in the presence of a high (10 mM) or low (2 mM) glucose concentration, by measuring cell density for four days after treatment (every 2 days), with an increasing concentration of BRAFi (vemurafenib). The IC50 (M) was calculated using GraphPad (PRISM10.0^®^). Statistical analysis (using unpaired *t*-test) has been performed between the mean of 4 independent experiments at different concentrations; *p* < 0.01 **; *p* < 0.001 ***. **F** SKS or SKR invalidated or not for AhR (KO) or treated with AhR-specific inhibitor (CH223191, 10 μM) were grown in increasing concentration of glucose (1, 2, and 10 mM). AhR and MITF protein levels were analyzed in these cells by western blotting. **G** Expression heatmap for various gene signatures established by the median of expression for specific genes from RNAseq data from SKS and SKR, KO or not for AhR and grown in increasing concentration of glucose (1 and 10 mM). Genes and clusters with similar expression profiles across the cohort are placed close to each other in the grid. The scale corresponds to the Z scores. **H** Expression heatmap (results from qPCR) for various glycolytic genes after or not KD of both AhR and MITF transcription factor by siRNA (siAhR#1-2, siMITF#1-2). Genes and clusters with similar expression profiles across the cohort are placed close to each other in the grid. The scale corresponds to the Z scores.

To investigate the role of AhR in metabolic rewiring of BRAFi-resistant cells, we quantified the glycolytic rate (termed glycoPER) and oxygen consumption (OCR) using Seahorse technology in BRAFi-sensitive (SKS) and resistant (SKR) cell lines, with or without AhR transcription factor (AhR knockout using CRISPR-Cas9 technology; Appendix Fig. S1A, B). Seahorse metabolic flux analysis revealed a significant reduction in glycolysis in SKR cells compared to that in SKS cells, accompanied by an increase in the rate of oxygen consumption (OCR), indicative of an increase in mitochondrial OXPHOS (Fig. 1A). Consequently, the culture medium of SKR cells exhibited reduced acidity, lower lactate concentrations, and higher levels of glucose and pyruvate (Fig. 1B). The absence of AhR had a comparable effect on BRAFi-sensitive and resistant cells, decreasing glycolysis and increasing OXPHOS (Fig. 1A). Accordingly, the analysis of glycolysis-associated signatures (Supplementary Table 2) in RNA sequencing data showed that glycolysis-pathways were significantly downregulated at the transcriptomic level in BRAFi-resistant cells and that AhR loss lowered glycolysis (Fig. 1C). These transcriptomic signatures were supported by data from BRAFi-resistant cell lines (obtained from the CCLE) [33] and dedifferentiated melanoma cell lines [19] (Fig. 1D). Importantly, glucose deprivation (1–2 mM vs 10 mM) rendered SKS cells less sensitive to BRAFi and SKR cells more resistant independent of the presence of AhR (Fig. 1E). Given the well-established association between the expression and activity of the transcription factors MITF and AhR and the molecular and metabolic sensitivity and plasticity of melanoma cells, we investigated their expression levels in response to different glucose concentrations in the culture medium. High MITF expression was observed in BRAFi-sensitive melanoma cells (green), whereas resistant cells no longer expressed MITF (Fig. 1F and Appendix Fig S1C). In contrast, AhR protein expression remained relatively stable across the different cell lines. The mRNA expression levels of AhR and MITF appeared to be inversely correlated in melanoma cell lines (*n* = 77, Depmap) (Appendix Fig S1D). Glucose starvation (1–2 mM vs. 10 mM) significantly decreased MITF protein expression in SKS cells, whereas AhR protein expression levels remained unchanged in both SKS and SKR cells (Fig. 1F and Appendix Fig S1E). Knockout of AhR or inhibition of its activity by the specific inhibitor CH-223191 (10 μM) did not affect MITF protein expression in response to glucose conditions (Fig. 1F). Similar results were observed in the 501Mel melanoma cell line, which more strongly expressed MITF (Appendix Fig S1E, F). Furthermore, analysis of RNA sequencing expression data revealed that in SKS glucose deprivation significantly reduced the expression of genes associated with glycolysis (Fig. 1G and Appendix Fig S1G). Accordingly, depletion of MITF by RNA interference (siRNA) in SKS (Fig. 1H) resulted in a significant decrease in the expression levels of genes involved in glucose metabolism, whereas depletion of AhR had a minimal impact on their expression (Fig. 1G and Appendix Fig S1G). In conclusion, our findings recapitulate a decrease in MITF expression in SKR lines and in glucose deprivation conditions leading to a reduction in the glycolysis rate; and found that AhR is required for oxygen consumption, favoring OXPHOS in BRAFi-resistant cells.

### Mitochondrial activity and ROS accumulation in BRAFi-resistant cells

**Fig. 2:**
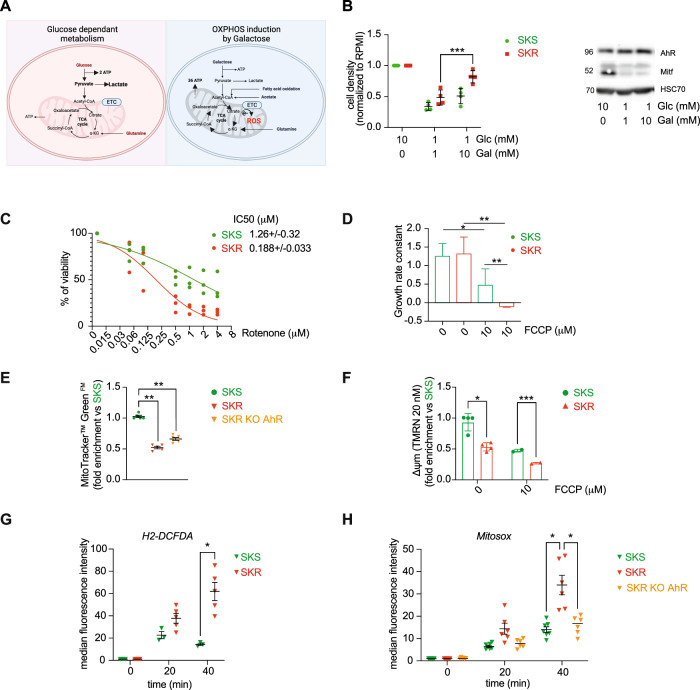
Mitochondrial and OXPHOS activity, and ROS accumulation in BRAFi-resistant cells. **A** Schematic representation of Glycolytic and OXPHOS metabolisms in the presence of glucose or galactose. **B** Measurement of the cell density of SKS and SKR cells cultured under conditions of glucose deprivation and supplemented or not with galactose (10 mM). Statistical analysis (using unpaired t-test) has been performed compared to the culture condition with a high concentration of glucose (10 mM) (mean + /sem; *n* = 4; *p* < 0.01 **; *p* < 0.001 ***. AhR and MITF protein levels were analyzed in these cells by western blotting. **C** Measurement of cell viability of SKS and SKR in the presence of increasing concentrations of Rotenone (inhibitor of complex I of the mitochondrial respiratory chain). The IC50 (M) was calculated using GraphPad (PRISM10.0^®^) (*n* = 3). **D** SKS and SKR were grown in the presence or absence of the uncoupler of mitochondrial oxidative phosphorylation FCCP (6 μM) for 4 days. Growth rate constant was calculated for four independent experiments (*p* < 0.05*; *p* < 0.01**; *p* < 0.001***). **E** Measurement of mitochondrial mass in SKR and R-KO compared to SKS after labeling with the fluorescent marker MitoTracker™ Green^FM^ (mean + /sem; *n* = 4; unpaired t-test; *p* < 0.01**). **F** Measurement of mitochondrial membrane potential (Δψm) in SKS and R after labeling with the fluorescent marker TMRM (20 nM final concentration, 30 min in dark at 37 °C) under basal conditions or after treatment with FCCP (10 μM) (*n* = 4; unpaired t-test *p* < 0.05*; *p* < 0.01**; *p* < 0.001***). **G** Measurement of the accumulation (0, 20, and 40 min) of total cellular ROS (reactive oxygen species) in SKS and R after labeling with the fluorescent marker CM-H2DCFDA (5 μM) (mean + /sem; *n* = 3, 4; unpaired t-test *p* < 0.01**). **H** Measurement of the accumulation (0, 20, and 40 min) of mitochondrial ROS (reactive oxygen species) in SKS, R, and R-KO after labeling with the fluorescent marker MitoSOX™ (5 μM) (mean + /sem; *n* = 3, 4; unpaired t-test *p* < 0.01**).

Next, we sought to characterize the mitochondrial activity of BRAFi-resistant melanoma cells by examining their dependency to TCA cycle and ATP production. To this end, we exposed cells to galactose instead of glucose. Indeed, the conversion of glucose to pyruvate through glycolytic metabolism yields 2 net ATP, whereas the conversion of galactose to pyruvate does not, necessitating an increased reliance on OXPHOS for energy production (36 ATP) (Fig. 2A). As anticipated, SKR cells exhibited an elevated dependency on OXPHOS compared to glycolysis, as SKR glucose-starved cells supplemented with galactose were able to resume proliferation independently of MITF expression (Fig. 2B). In addition, SKR cells exhibited increased sensitivity to rotenone, an inhibitor of mitochondrial respiratory chain complex I (Fig. 2C) and their growth was severely inhibited by the mitochondrial OXPHOS uncoupler carbonyl cyanide 4-(trifluoromethoxy)phenylhydrazone (FCCP), further supporting their dependency on OXPHOS (Fig. 2D). To validate the difference in mitochondrial activity between SKS and SKR cells, we measured the mitochondrial mass using MitoTracker™ Green FM Dye (Thermo Fischer Scientific) (Fig. 2E) and the changes in mitochondrial membrane potential (Δψm) using the fluorescent dye tetramethylrhodamine methyl ester (TMRM) (Fig. 2F) via flow cytometry. SKR cells exhibited significantly lower mitochondrial mass (Fig. 2E) and lower membrane potential (Fig. 2F) than SKS cells did. In addition, given that highly fused mitochondria are associated with elevated OXPHOS activity under stress, we analyzed mitochondrial morphology in cells [34]. Labeling with MitoTracker Green revealed that SKS mitochondria were significantly fragmented, whereas SKR and SKR-KO AhR mitochondria appeared hyperfused supporting the reliance of SKR cells on OXPHOS (Appendix Fig. S1H).

Electrons can escape from the electron transport chain during both normal electron transport and mitochondrial dysfunction, leading to the formation of superoxide anions through one-electron reduction of oxygen. This process indicates that mitochondria can significantly contribute to the generation of cellular ROS under certain conditions. To investigate this phenomenon, we compared the levels of both total and mitochondrial ROS in BRAFi-sensitive (SKS) and resistant (SKR) cells using 5-(and-6)-chloromethyl-20,70-dichlorodihydrofluorescein diacetate (CM-H2DCFDA) and MitoSOX probes, respectively. The results revealed that both total (Fig. 2G) and mitochondrial (Fig. 2H) ROS levels were significantly higher in the SKR cells than in the SKS cells. Importantly, the absence of AhR in SKR cells significantly decreased the level of mitochondrial ROS, which was comparable to that in SKS (Fig. 2H). This observation is in accordance with established knowledge that AhR activation by its ligands can promote ROS formation in cells [35].

### Induction of ferroptosis in BRAFi-resistant melanoma cells

**Fig. 3:**
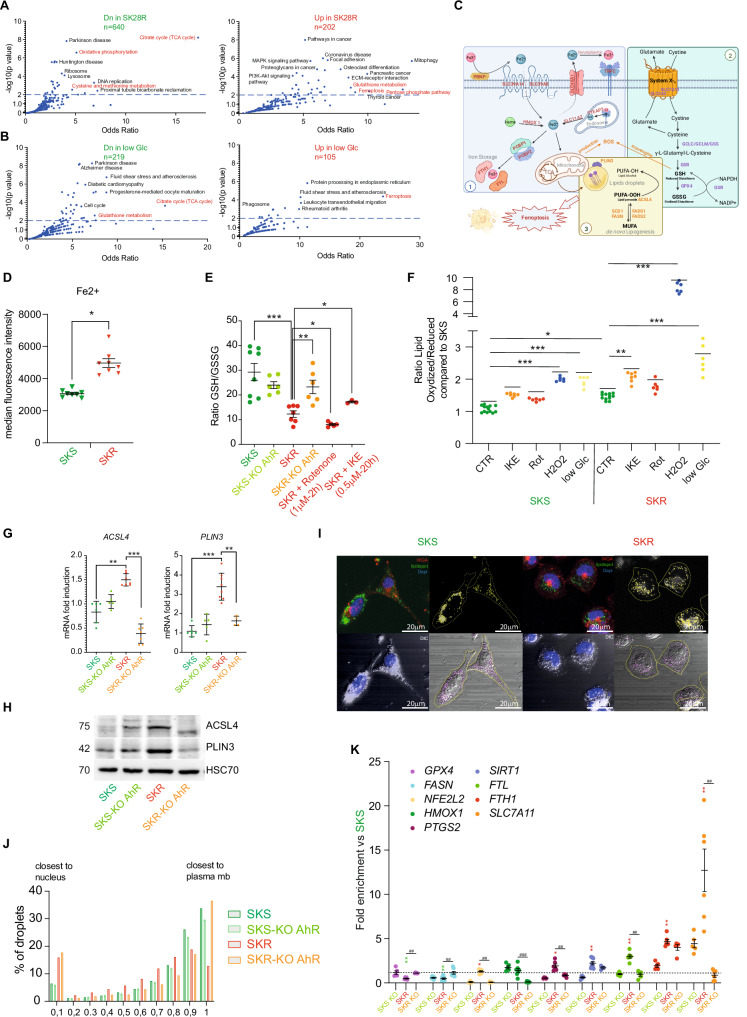
Induction of ferroptosis pathway in BRAFi resistant cells. **A** Volcano plot (using GraphPad (PRISM10.0^®^) of terms from the KEGG_2021_Human gene set plotted by the corresponding odds ratio (*x*-position) and −log10 (*p*-value) (y-position) from the enrichment results in SKS (middle) or in SKR (right). **B** Volcano plot (using GraphPad (PRISM10.0^®^)) of terms from the KEGG_2021_Human gene set plotted by the corresponding odds ratio (*x*-position) and −log10 (*p*-value) (*y*-position) from the enrichment results for down-regulated genes in low Glc (middle) or up-regulated genes in low Glc (right). **C** Schematic representation of activation of ferroptosis in cell. The figure shows the regulatory pathways of ferroptosis, which can be roughly divided into three categories. The first one is the regulation mechanism of iron metabolism (1). The second mechanism is regulated by GSH/GPX4 pathway, such as inhibition of system Xc-, glutamine pathway (2). The third category is related pathways around lipid metabolism and lipid peroxidation of PUFA (3). All these regulatory pathways are closely related to cell metabolism and the accumulation of oxidative stress in cells. **D** Measurement of Fe2+ level in SKS and SKR after cell labeling with BioTracker™ FerroOrange Live Cell Dye (Merck) (mean + /sem; *n* = 8; *p* < 0.05*). **E** Measurement of ratio reduced Glutathione (GSH)/oxidized Glutathione (GSSG) in SKS, SKS KO, SKR and SKR KO at basal conditions. Treatment of SKR with Rotenone (0.5 μM) or IKE (0.5 μM) was used as positive control for the accumulation of GSSG (mean + /sem; *n* = 5–7; *p* < 0.05). **F** Measurement of reduced and oxidized lipids in SKS and SKR after cell labeling with BODIPY™ (581/591) C11. Treatment with Rotenone (0.5 μM), IKE (0.5 μM), H2O2 (0.8 μM), and low glucose medium was used to see the impact on oxidation of lipids in response to treatment (mean + /sem; *n* = 4–6; *p* < 0.05*; *p* < 0.01**; *p* < 0.001***). **G** *ACSL4* and *PLIN3* mRNA expression was measured by RT-qPCR in SKMel28 cells. Statistical analysis using unpaired *t*-tests method has been performed to compare the level of expression compared to SKS, *p* < 0.01**; *p* < 0.001***. **H** ACSL4 and PLIN3 protein levels in SKMel28 cells were analyzed by western blotting. **I** Confocal microscopic analysis (Confocal Microscope Leica TCS SP8 with AOBS and Resonant Scanner (8 kHz), ×40) of lipid droplets (LD) in SKS and SKR after labeling with the fluorescent probe LIPIDSPOT-610 (Interchim, B30VJ1) (upper panel) or visualized directly by DIC (Differential Interference Contrast microscopy) (lower panel). Labeling with the fluorescent probe WHEAT GERM AGGLUTININ-CF488 (Interchim, AQK230) and DAPI was used to delimit plasma membrane and nucleus in cells. **J** Lipid droplets sub-cellular distribution in SKS and SKR cells. The ratio between the distance to the nucleus with the sum of the distance to the nucleus + the distance to the plasma membrane has been calculated for each LD (number of cells >150, number of LD/cell 50-100). **K** Graph representing the variations in expression of genes for different markers of ferroptosis (http://www.zhounan.org/ferrdb/current/) in SKS KO AhR, SKR, SKR KO AhR compared to SKS (mean + /sem; *n* = 4–6; *p* < 0.05*^, #^; *p* < 0.01**^, ##^; *p* < 0.001***^, ###^).

To elucidate the underlying mechanisms supporting the metabolic changes associated with resistance to BRAF inhibitors, we established an mRNA signature (RNA-seq) of SKS and SKR cells under normal culture conditions and after glucose deprivation (Appendix Fig. S2A, B). Interestingly, functional analysis (EnrichR) of differentially expressed genes between SKS and SKR cells revealed upregulation of genes related to glutathione metabolism, the pentose phosphate pathway, and ferroptosis in SKR cells (Fig. 3A). Similarly, glucose deprivation increased the expression of ferroptosis-associated genes (Fig. 3B). Ferroptosis is a type of cell death caused by iron-dependent lipid peroxidation that is often associated with various metabolic disorders and disrupted homeostasis (Fig. 3C). Unlike apoptosis, necrosis, and other cell death mechanisms, ferroptosis is distinctly regulated.

Given that elevated iron levels in the labile iron pool (LIP) are the primary drivers of the Fenton reaction and lipid peroxidation, we measured the levels of reduced iron (Fe^2+^) in SKS and SKR cells using the BioTracker™ FerroOrange Live Cell Dye (Merck). We found a significant increase in Fe^2+^ levels in SKR cells compared to SKS cells (Fig. 3D). This increase in Fe^2+^ levels in BRAFi-resistant cells could be attributed to elevated expression of protein involved in iron homeostasis including the divalent metal ion transporter 1 (DMT1, also known as solute carrier family 11 member 2, SLC11A2), TFRC and PRNP ion transporter, and the iron storage ferritin complex (FTL and FTH1) (Appendix Fig. S2C). Moreover, subjecting SKMel28 cells to glucose starvation (1–2 mM vs. 10 mM) significantly upregulated the expression of these genes (Appendix Fig. S2C).

Ferroptosis induction is linked to the inactivation or reduction of glutathione peroxidase 4 (GPX4) production using glutathione (GSH) as a cofactor to resist lipid peroxidation (Fig. 3C2). To investigate this aspect, we measured the levels of reduced (GSH) and oxidized (GSSG) glutathione in both SKS and SKR cell lines, with or without AhR, using a colorimetric assay (ElabSciences) (Appendix Fig. S2D). We found that GSSG levels were significantly higher in SKR cells than in SKS cells, (Appendix Fig. S2D), leading to a significant decrease in the reduced-to-oxidized glutathione ratio (GSH/GSSG) in SKR cells (Fig. 3E). This decreased ratio reduces the pool of GSH available to counteract intracellular ROS, favoring lipid peroxidation. Importantly, the loss of AhR in SKR cells, increased the GSH/GSSG ratio to a level comparable to that in SKS cells (Fig. 3E). This finding aligns with previous observations of the reduced to oxidized GSH/GSSG ratio in B16 melanoma cells following AhR activation by canonical ligands such as BaP or FICZ [35]. Hence, it appears that BRAFi-resistant cells accumulate more oxidized glutathione (GSSG) and less reduced glutathione (GSH), which correlates with AhR activity. This observation was further supported by the upregulation of enzymes involved in glutathione metabolism, particularly following glucose deprivation in the culture medium (Appendix Fig S2E).

Previous studies have demonstrated that the BRAF^V600E^ mutation promotes the accumulation of long-chain polyunsaturated fatty acids (PUFAs), with non-responder cohorts of melanoma patients showing significantly increased levels of immunomodulatory lipids and long-chain fatty acids [36]. To deepen our understanding of these findings, we analyzed metabolomic data from the Cancer Cell Line Encyclopedia (CCLE) (DepMap) and confirmed that long-chain PUFAs were significantly enriched in BRAFi-resistant cells (Appendix Fig. S2F). This enrichment correlated with decreased expression of stearoyl-CoA desaturase 1 (SCD1) and fatty acid synthase (FASN), which promote monounsaturated fatty acids (MUFAs) in resistant cells, especially after glucose deprivation (Appendix Fig. S2G).

Given that, abnormal accumulation of ROS, resulting from disequilibrium in scavenging factor systems including glutathione peroxidase (GPX4), glutathione reductase (GR), superoxide dismutase (SODs), and catalase leads to the peroxidation of PUFAs and promotes ferroptosis. We assessed lipid peroxidation levels using BODIPY™ C11 labeling after treatment with the strong oxidative agent H_2_O_2_, the ferroptosis inducer Imidazole Ketone Erastin (IKE), rotenone, or glucose deprivation. The results indicated higher lipid peroxidation in SKR cells than in SKS cells under basal conditions and a significant effect after inducer treatment or glucose deprivation (Fig. 3F). Acyl-CoA synthetase long-chain family member 4 (ACSL4), which is a crucial determinant of ferroptosis sensitivity by catalyzing PUFAs biosynthesis and promoting lipid peroxidation accumulation, was significantly upregulated at RNA and protein levels in SKR cells. This increase was reversed in the absence of the AhR transcription factor (Fig. 3G, H). Accordingly, AhR activation with various canonical ligands (ITE (5 μM), TCDD (10 nM), BaP (5 μM), FICZ (5 μM), and Indirubin (5 μM)) in SKR cells (24 h) induced significantly ACSL4 and PLIN3 protein expression (Appendix Fig S3A). Together, this confirms the role of AhR in lipid peroxidation regulation and ferroptosis induction.

Lipid droplet (LD) formation via diacylglycerol O-acyltransferase (DGAT)-mediated triglyceride synthesis can act as a sink for phospholipid-derived PUFAs, thereby preventing their peroxidation. LD biogenesis may also restrict lipid peroxidation by sequestering already damaged peroxidized PUFAs. Our analysis revealed an important shift in LD subcellular localization closer to the nucleus in SKR cells (Fig. 3I, J and Appendix Fig. S3B) and a significant decrease in LDs in SKR cells (Appendix Fig. S3C). In the absence of AhR, the distribution and quantity of LD in the SKR were comparable to those in the SKS (Fig. 3I, J and Appendix Fig. S3B, C). Perilipin 3 (PLIN3), a key player in LD biogenesis and lipolysis, was significantly upregulated in SKR cells, an effect that was also AhR-dependent (Fig. 3G, H). However, this upregulation did not correlate with an increase in LD number in SKR, but was associated with a significant modification of LD subcellular localization as observed after its depletion (Appendix Fig. S3D–F). In summary, our findings indicate that BRAFi-resistant cells exhibit increased intracellular or mitochondrial ROS, decreased of GSH levels, elevated long-chain PUFAs, and enhanced lipid peroxidation. Moreover, SKR cells show differences in LD number and subcellular localization, with a distribution closer to the nucleus. These cellular alterations contribute to the elevated susceptibility of BRAFi-resistant cells to ferroptosis, a phenomenon that is largely regulated by AhR. Depletion of AhR reversed this phenotype, possibly through regulation of PLIN3 and ACSL4 expression (Fig. 3G, H).

To further validate the activation of the ferroptosis pathway in the SKR and the underlying role of AhR, we examined the expression levels of several genes defined as positive markers of ferroptosis (*FTL*, *FTH1*, *SLC7A11*, *HMOX1*, etc.) sourced from the FerrDb database, a repository dedicated to ferroptosis regulators and markers (http://www.zhounan.org/ferrdb/current/) [37]. The analysis revealed a significant enrichment of positive ferroptosis markers in SKR cells (*NFE2L2*, *PTGS2*, *SIRT1*, *FTL*, *FTH1*, and *SLC7A11*) coupled with a reduction in the expression of negative markers (*GPX4*, *FASN*), confirming the activation of ferroptosis (Fig. 3K). Notably, the absence of AhR transcription factors in SKR cells also led to downregulation of ferroptosis marker expression (Fig. 3K). This was supported by the analysis of gene expression of various ferroptosis drivers (http://www.zhounan.org/ferrdb/current/) in BRAFi-sensitive (green) or resistant (red) melanoma cells from the Cancer Cell Line Encyclopedia (CCLE) or from our laboratory, which demonstrated an enrichment of these genes in resistant cells, as well as in dedifferentiated melanoma cells [19] (Appendix Fig. S4A–D). Consistently, we observed upregulation of several ferroptosis drivers in SKR cells compared to SKS cells (Appendix Fig. S4E), and this induction was attenuated in SKR cells lacking AhR. Utilizing the CellMiner database across multiple cancer cell lines, we found that the ferroptosis inducer Erastin exhibited significantly greater efficiency (IC50) in cells expressing high levels of ferroptosis markers and driver genes (Z-score) (Appendix Fig. S4F). Additionally, volcano plots illustrated a significant positive correlation (*p* < 0.001) between AhR expression levels and Erastin sensitivity (Appendix Fig. S4F). Together, these results support the role of the AhR transcription factor as an important regulator of genes involved in ferroptosis, suggesting a key role for AhR in promoting ferroptosis susceptibility.

### BRAFi-resistant cells are more sensitive to ferroptosis inducers

**Fig. 4:**
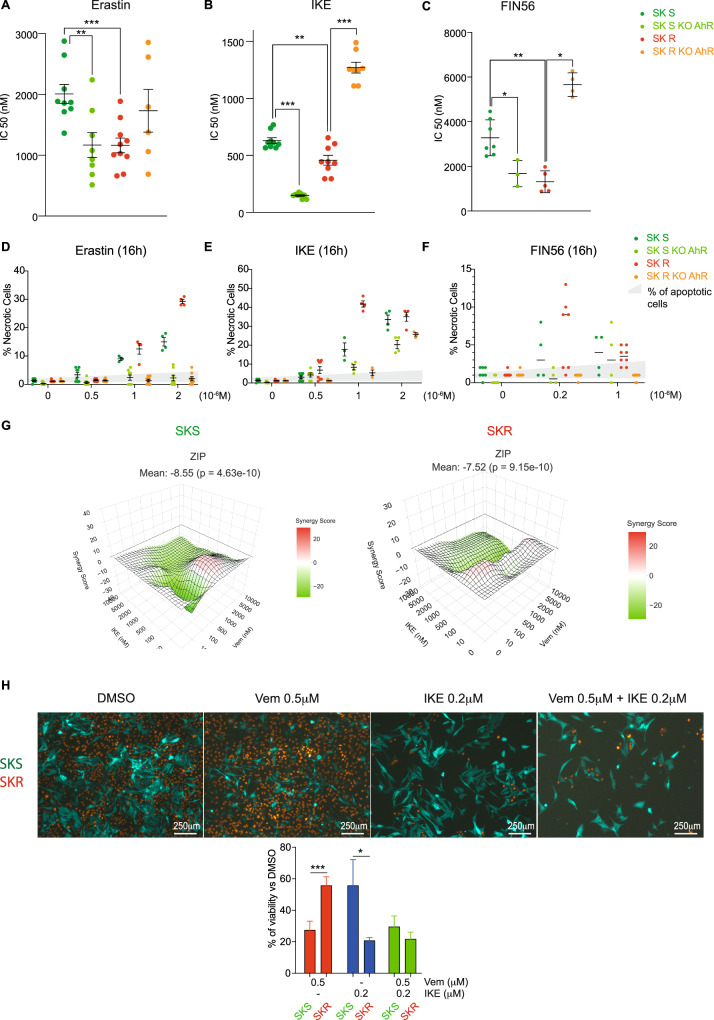
BRAFi-resistant cells are more sensitive to ferroptosis inducers. Erastin (**A**), IKE (**B**), and FIN56 (**C**) sensitivity was established in SKS KO AhR, SKR, SKR KO AhR, by measuring cell density for four days after treatment (every 2 days), with an increasing concentration of ferroptosis inducers. The IC50 (nM) for each experiment was calculated using GraphPad (PRISM10.0^®^) (mean + /sem; *n* = 9; *p* < 0.05*; *p* < 0.01**; *p* < 0.001***). SKS and SKR cells were treated for 16 h with increasing concentrations of inducers of ferroptosis, Erastin (**D**), IKE (**E**), and FIN56 (**F**). At the end of the treatment, cells were stained with Hoechst, propidium iodide, and YO-PRO^®^−1 to evaluate the percentage of apoptotic (represented in red) and necrotic (represented in blue) cells relative to the number of Hoechst-positive cells by microscopy (mean + /sem; *n* = 4, 5). **G** Synergy map (Synergy finder+) obtained from combined treatment for 4 days of SKS and SKR cells with increasing concentrations of BRAFi (Vemurafenib) and ferroptosis inducer (IKE). **H** Microscopic analysis (Axio Vert.A1 inverted microscope (Carl Zeiss) at ×5 magnification) of SKS (GFP, green) and SKR (dTomato, red) after 4 days of treatment (every 2 days) with vehicle (DMSO), Vemurafenib (0.5 μM) alone, IKE (0.2 μM) alone or with the combination (Vem, IKE). Quantification of residual cells has been performed after counting of cells and analysis by flow cytometry.

Since BRAFi-resistant cells exhibit increased ferroptosis activation capacity compared to sensitive cells, we measured the impact of various ferroptosis inducers on these cells. We determined the IC50 for three ferroptosis activators, Erastin, IKE and FIN56, and found that SKR cells were significantly more sensitive to ferroptosis inducers than SKS cells (Fig. 4A–C).

In order to definitively assess that the cell death induced in SKR is indeed ferroptosis, we co-treated SKR cells with IKE at their respective IC50 doses in the presence or absence of different ferroptosis inhibitors (UAMC-3203; Ferrostatin-1, and Liproxstatin-1). Importantly, the concomitant use of ferroptosis inhibitors significantly counteracts the IKE-induced cell death of SKR cells, demonstrating that the observed cell death is ferroptosis (Appendix Fig. S5A). Similar effects were observed in two additional pairs of BRAFi-sensitive and -resistant melanoma cell lines (M229S/R and M238S/R, respectively) (Appendix Fig. S5B). Further validation confirmed that SKR cells were indeed significantly more prone to ferroptosis than SKS cells, as evidenced by the elevated levels of necrotic cells after 16 h of treatment with ferroptosis inducers (Erastin, IKE, and FIN56) **(**Fig. 4D-F**)**. Intriguingly, the absence of AhR in both SKS and SKR cells prevented necrosis activation by ferroptosis inducers (Erastin, IKE, FIN56). These findings demonstrate the higher sensitivity of BRAFi-resistant melanoma cells to ferroptosis inducers, opening avenues for novel therapeutic strategies alongside targeted therapies. However, we did not observe any synergistic effect between the ferroptosis inducer (IKE) and the BRAF inhibitor (Vem) using combinations of increasing concentrations of the inhibitors (Fig. 4G). To illustrate the distinct responses of BRAFi-sensitive and -resistant cells to targeted therapy and ferroptosis inducers, we treated a 50/50% mixture of SKS (constitutively expressing GFP in green) and SKR (constitutively expressing dTomato in red) for 4 days with BRAFi (Vemurafenib, 0.5 μM), IKE (0.2 μM), or both. Subsequently, we measured the residual cells using fluorescence microscopy and quantified them using flow cytometry. The mortality of SKS following Vemurafenib treatment was significantly higher than that of SKR (Fig. 4H, red). Conversely, IKE treatment eradicated SKR but not SKS (Fig. 4G, blue). Combining Vemurafenib with IKE induced mortality in both SKS and SKR cells (Fig. 4G, green), highlighting the potential synergistic effects of targeting both pathways.

**Fig. 5:**
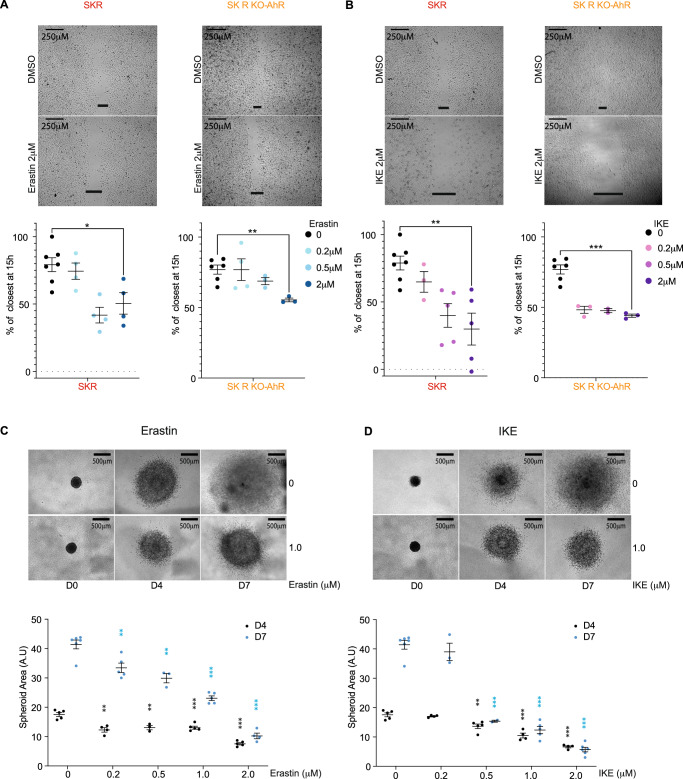
Ferroptosis inducers prevent migration and invasion of BRAFi-resistant cells. Wound-healing assays were performed using IBIDI^®^ chambers to evaluate the role of inducers of ferroptosis on cell migration in SKR (**A**) and SKR KO (AhR) (**B**) treated with increasing concentrations of Erastin or IKE (0.2, 0.5 and 2 µM). Images of the wound were acquired at a ×5 magnification with an Axio Vert.A1 inverted microscope (Carl Zeiss). The histogram shows the mean wound closure determined by measuring the distance between the edges of the wound at time 0 and 15 h (mean + /sem; *n* = 4–7; *p* < 0.05 *; *p* < 0.01 **; *p* < 0.001 ***). Scale bar 250 µm. Three-dimensional spheroid growth of SKR (**C**) and SKR KO (AhR) (**D**) after treatment every 2 days with the inducers of ferroptosis Erastin or IKE (1 μM). Images were captured four days and seven days after implantation of the spheroids into collagen gel. Analysis of invasive capacity was measured by comparing the area of the final spheroid compared to the initial one. (mean + /sem; *n* = 3–7; *p* < 0.05*; *p* < 0.01**; *p* < 0.001***). Scale bar 500 µm.

SKR cells exhibited elevated migration and invasive capabilities compared to SKS cells. To explore the potential of ferroptosis inducers (Erastin and IKE) to mitigate cell migration, we conducted wound-healing assays on SKR cells with or without genetic depletion of AhR. Treatment with increasing concentrations of Erastin (Fig. 5A) or IKE (Fig. 5B) significantly attenuated the migration of SKR cells. The absence of AhR slightly mitigated the efficacy of ferroptosis inducers in limiting cell migration (Fig. 5A, B). Subsequently, we assessed the invasive properties of SKR cells using tumor-spheroid assays, which mimic the 3D architecture of melanoma, in the presence or absence of ferroptosis inducers (Erastin or IKE). Erastin (Fig. 5C) and IKE (Fig. 5D) treatments significantly suppressed the invasive capacity of SKR cells at both 4 and 7 days.

### BRAFi-resistant tumors are sensitive to ferroptosis inducers

**Fig. 6:**
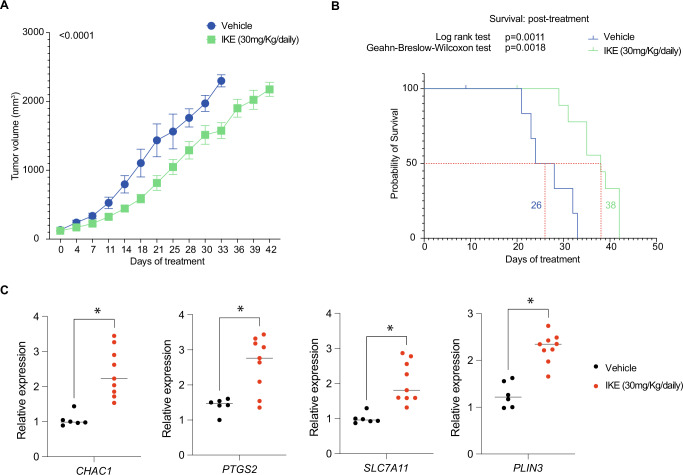
Induction of ferroptosis by IKE delays tumor growth of BRAFi-resistant PDX melanoma. **A** PDX model MEL006R (BRAFi resistant) was randomly implanted in NMRI nude mice. Mice with tumors reaching 200 mm³ were treated daily with IKE (Das, Selleckchem, 30 mg/kg, *n* = 9) or with vehicle DMSO (*n* = 6). PDX tumor volumes were measured every three days until reaching 2000 mm^3^. Values correspond to mean ± sem. Statistical analysis (two way ANOVA) was performed between different experiments (IKE vs CTR). **B** Kaplan–Meier survival curve for MEL006R mice treated with the different drugs. Survival curves have were compared using the nonparametric log-rank (Mantel-Cox) test. **C** Analysis of the expression levels of different ferroptosis-related genes from PDX tumors (at final time point : 30–40 days) treated or not by IKE (30 mg/Kg) related to Fig. 6 (mean + /sem; *n* = 9; *p* < 0.001).

These in vitro evidences prompted us to examine the clinical relevance of using ferroptosis inducers to treat BRAFi-resistant tumors. To this end, we used the Mel006R BRAFi-resistant Patient-Derived Xenograft (PDX) mice model (Paris et al. [30]). BRAF^V600E^ cutaneous melanoma PDX MEL006R is derived from MEL006 PDX lesions at relapse [38] upon the acquisition of resistance to BRAFi/MEKi treatment (Dabrafenib and Trametinib). Once the tumors reached 100 mm^3^, grafted mice were treated with or without IKE at a final concentration of 30 mg/kg/daily (ferroptosis inducers). It was noted that any toxicity of the drug (the weight of the mice was stable during treatment (Appendix Fig. S6A)) and no other side effects were noted. Tumor growth was monitored every three days until the tumor reached 2000 mm^3^. IKE significantly slowed down tumor growth (Fig. 6A and Appendix Fig. S6B) and the mice treated with ferroptosis inducers survived significantly longer (MS: 38 days) than those treated with vehicle alone (MS: 26 days) (Fig. 6B). To assess the induction of ferroptosis after treatment of PDX tumors with IKE, we measure at end point the expression levels of a set of ferroptosis markers (*SLC7A11, CHAC1, PTGS2, PLIN3*) [39] and found a significant increase in the expression of ferroptosis markers in the IKE-treated PDX group compared to the control group (Fig. 6C). Together, this reinforces the role of ferroptosis inducers as an escape route to cure BRAFi-resistant melanoma. In conclusion, our findings demonstrate that treatment with ferroptosis inducers such as IKE holds promise for targeting BRAFi-resistant cells and mitigating their aggressive phenotype.

## Discussion

Our study revealed that drug-resistant cells exhibit elevated susceptibility to ferroptosis, a process that we show to be dependent on the AhR pathway and that has gained significant attention in recent years [40, 41].

We first demonstrated that BRAFi resistance correlates with upregulation of mitochondrial energy metabolism (OXPHOS), accumulation of ROS, and disturbances in lipid peroxidation, storage, and iron and glutathione metabolism (Graphical Abstract). These metabolic alterations in resistant cells culminate in a heightened predisposition to ferroptosis activation. Second, we revealed the role played by AhR, along with the MITF transcription factor in mediating the metabolic reprogramming.

Notably, Wang et al. demonstrated that deficiencies in Wnt/β-catenin signaling exacerbate ferroptosis in melanoma by regulating MITF via the downregulation of PGC1α and SCD1, leading to suppressed lipid peroxidation and ultimately inhibiting ferroptosis [42]. Consistently, our findings align with the observed decrease in MITF expression in BRAFi-resistant cells or under glucose starvation conditions, showing its pivotal role in orchestrating metabolic rewiring, such as the TCA cycle [27], SCD1-mediated fatty acid [8], and the oxidative stress response [43]. Our study further revealed that these metabolic shifts are not solely due to MITF loss but also to the upregulation and activation of AhR. Similar to the phenotype-switching model, where a high MITF^low^/AXL^high^ indicates an invasive and resistant phenotype [20, 44], we observed a comparable balance with AhR. Elevated AhR expression and activity in the absence of MITF in melanomas correlates with an OXPHOS phenotype, intracellular ROS accumulation, lipid peroxidation of PUFAs, and dysregulated glutamine metabolism. Moreover, we have implicated AhR in the regulation of various inducers and markers of ferroptosis. Despite limited prior investigations into the role of AhR in ferroptosis activation, Cui et al. demonstrated that the canonical AhR-ligand L-kynurenine induces NK cell loss in the gastric cancer microenvironment by promoting ferroptosis [45]. AhR activation during reoxygenation under ischemia-reperfusion conditions induces ROS production, lipid peroxidation, and ferroptotic cell death [46]. Additionally, Kwong et al. proposed a nuclear receptor meta-pathway (NRM) model, illustrating a close association between NRF2 and AhR expression levels and susceptibility to the ferroptosis inducer Erastin [47]. Tight regulation of AhR and NRF2 expression and activity is crucial for controlling ferroptosis in melanoma [48, 49].

We demonstrated that targeting ferroptosis presents a promising therapeutic avenue to combat resistance to targeted therapy in melanoma. Tsoi et al. suggested the use of ferroptosis inducers, such as Erastin, to deplete the pool of persistent dedifferentiated melanoma cells when combined with BRAFi [19]. In addition, we elucidated the transcriptional and metabolic mechanisms underlying ferroptosis induction, highlighting the pivotal role of the AhR transcription factor. Recent studies have focused on identifying novel strategies to enhance therapeutic efficacy when combined with targeted melanoma therapies, particularly those targeting the specific metabolic pathways of resistant cells to induce ferroptosis. Sorafenib sensitizes melanoma cells to vemurafenib by increasing ROS production via ferroptosis [50]. Gambogenic acid (GNA), a xanthone found in Gamboge, induces ferroptosis in TGF-β1-stimulated melanoma cells through the p53/SLC7A11/GPX4 signaling pathway by upregulating p53 expression [51].

Combining ferroptosis inducers with targeted therapies or immune checkpoint inhibitors can potentially increase patient response rates. Emerging ferroptosis inducers, such as erastin, RSL3, ML162, ML210, JKE-1674, FIN56, and imidazole ketone erastin (targeting dedifferentiated melanoma cells), are being considered for clinical trials in combination with MEK and BRAF inhibitors, although such trials have yet to commence in melanoma. Comparable strategies have also been developed to address acquired resistance to therapy. Talebi et al. recently targeted lipid poly-unsaturation in therapy-resistant models by combining MAPK inhibition with the preclinical FASN inhibitor TVB-3664, which attenuated cell proliferation and sensitized cells to various ROS inducers [52]. Additionally, Vergani et al. demonstrated that drug-resistant cells became more susceptible to BRAFi following lipid deprivation, and treatment with the SOAT inhibitor avasimibe increased their sensitivity to Vemurafenib by inhibiting cholesterol esterification [53].

In summary, our findings suggest that targeting ferroptosis through the modulation of AhR and associated metabolic pathways represents a promising strategy to mitigate resistance and improve melanoma therapeutic responses.

## Material and methods

### Cell culture and reagents

Human melanoma cell lines (SKMel28, 501Mel, M229, and M238) were grown in humidified air (37 °C, 5% CO_2_) in RPMI-1640 medium (Thermo Fisher Scientific, Invitrogen, Waltham, MA, USA) supplemented with 10% fetal bovine serum (Eurobio, Les Ulis, France) and 1% penicillin-streptomycin antibiotics (Thermo Fisher Scientific). SK28 (SK-MEL-28, RRID: CVCL_0526) (S + R) cells were obtained from J.C Marine at the VIB Center for Cancer Biology, VIB (Leuven, Belgium). M229 (RRID:CVCL_D748) and M238 (RRID:CVCL_D751) cells were obtained from Graeber’s laboratory at the UCLA Molecular Biology Institute (Los Angeles, CA, USA). SKMel28. 501Mel cells (S) were obtained from ATCC and 501Mel (501-Mel, RRID:CVCL_4633) BRAFi-resistant cells (R) were obtained after three months of treatment with Vem (1 μM every 2 days). No difference in proliferation was observed between resistant and parental cells. Melanoma cells were grown in the absence of BRAFi treatment but challenged every two weeks with BRAFi at the IC50 dose of the corresponding sensitive cells to maintain a selective pressure. All the cell lines were routinely tested for mycoplasma contamination.

### Reagents

#### BRAF inhibitors

- Vemurafenib (Vem, PLX4032) (Selleckchem, S1267, Houston, USA)

#### AHR inhibitor

- CH223191 (Selleckchem, S7711)

#### Ferroptosis inducers

- IKE (Imidazole Ketone Erastin (PUN 30119), Selleckchem, S8877)
- Erastin (Selleckchem, S7242)
- FIN56 (Selleckchem, S8254)

#### Ferroptosis inhibitors

- UAMC-3203 (Selleckchem, S8792)
- Ferrostatin-1 (Selleckchem, S7243)
- Liproxstatin-1 (Selleckchem, S7699)

#### Mitochondrial inhibitors

- Rotenone
- FCCP (MedChem Express, USA)

#### ROS inducers

- H2O2 (311421, Sigma Aldrich)

#### Fluorescent probes

- MitoTracker™ Green FM Dye (M7514, Thermo Fischer Scientific, USA)
- TMRM (Tetramethylrhodamine Methyl Ester) (T668, Thermo Fisher Scientific, USA)
- CM-H2DCFDA (5-(and-6)-chloromethyl-20,70-dichlorodihydrofluorescein diacetate) (C6827, Thermo Fisher Scientific, USA)
- MitoSOX™ Red Mitochondrial Superoxide Indicator (M36008, Thermo Fisher Scientific, USA)
- BODIPY™ 493/503 (4,4-difluoro-1,3,5,7,8-pentaméthyl-4-bora-3a,4a-diaza-s-indacène) (D3922, Thermo Fisher Scientific, USA)
- BODIPY™ 581/591 C11 (4,4-difluoro-1,3,5,7,8-pentaméthyl-4-bora-3a,4a-diaza-s-indacène) (D3861, Thermo Fisher Scientific, USA)
- *DMSO* (D8418, Thermo Fisher Scientific, USA).

### CRISPR/Cas9 experiments

AhR knockout was performed using CRISPR/Cas9 methodology. The guide sequence targeting AhR (Sigma-Aldrich, St Louis, MO, USA) was cloned into the GeneArt CRISPR Nuclease vector according to the manufacturer’s instructions (Life Technologies, Saint-Aubin, France). Next, 501Mel or SKMel28 cells were transfected with the vectors and the cells were seeded two days later in 96-well plates at 0.5 cells/well for single-cell clonal expansion. The clones of interest were validated by sequencing, western blot analysis, and RT-qPCR.

### RNA interference

siRNA transfection was performed using Lipofectamine RNAiMax (Thermo Fisher Scientific) following recommendations. Briefly, 2 × 10^5^ cells/well in 500 μl complete medium were plated in 6 wells dishes with a mix of 20 nM of siRNA and 1.5 μl of Lipofectamine RNAiMax (in 100 μl of OptiMEM, Thermo Fisher Scientific). Six hours after transfection, culture medium was changed. 24–72 h after transfection, cells were recovered for expression analysis or phenotype analysis. siRNA were purchased from IDT DNA: siCTR, siAhR, siMITF, and siPLIN3. Sequences of all siRNAs are available in Supplementary Table 1 (EV1).

### Cell density evaluation

Cell density was assessed using a methylene blue colorimetric assay. Briefly, cells were fixed for at least 30 min in 95% ethanol. Following ethanol removal, the fixed cells were dried and stained for 30 min with 1% methylene blue dye in a borate buffer. After four washes with tap water, the cells were dried and 100 μl 0.1 N HCl was added to each well. The plates were then analyzed using a spectrophotometer at 620 nm.

### Mitochondrial transmembrane potential (ΔΨ)

After incubation, the cells were detached, centrifuged (160 × g for 10 min) and suspended in 1 mL PBS plus TMRM (Tetramethylrhodamine Methyl Ester, 20 nM final concentration). The mixture was incubated in the dark at 37 °C for 30 min, centrifuged (160 × *g* for 10 min), and suspended in 300 μL of PBS. Fluorescence emission (548 nm) was detected using a FACS LSRFortessa™ X-20 Cell Flow Cytometer (BD Biosciences) including the BD FACSCanto™ Cell Analyzer, with 10,000 events per sample. Data analysis and graphs were generated using FlowJo vX.0.7 software (Ashland, USA, RRID:SCR_008520).

### ROS measurement

Cells were washed with PBS and resuspended at 10^5^ cells in 50 μL reaction volume 5-(and-6)-chloromethyl-20,70-dichlorodihydrofluorescein diacetate (CM-H2DCFDA) and MitoSOX (Thermo Fisher Scientific, USA) were used at a final concentration of 5 μM to measure cytoplasmic ROS levels and mitochondrial superoxide production. The cells were then incubated in the dark at 37 °C for 30 min. After release from ice at different time points (20 and 40 min), the fluorescence of CM-H2DCFDA and MitoSOX was measured at *λ*_em_ = 530 ± 30 nm and *λ*_ex_ = 489 ± 14 nm, respectively, on a LSRFortessa™ X-20 Cell Analyzer cytometer (BDbiosciences). Data were analyzed using the FlowJo® software.

### Dosage of intracellular glutathione

Total, oxidized, and reduced GSH levels in BRAFi-sensitive and resistant SKMel28 melanoma cells were quantified using a GSH kit (Elabscience, E-BC-K030-M) according to the manufacturer’s instructions [54].

### Dosage of lipid peroxidation

Oxidized (FITC) and reduced (PE) lipid levels in BRAFi-sensitive and resistant SKMel28 melanoma cells were quantified using BODIPY™ 581/591 C11 (Thermo Fisher Scientific, USA), respectively at a final concentration of 1 μM. Cells were incubated in the dark at 37 °C for 30 min and the fluorescence of reduced or oxidized lipids was measured at *λ*_ex_ = 489 ± 14 nm and *λ*_em_ = 565 ± 30 nm and, respectively, on an LSRFortessa™ X-20 Cell Analyzer cytometer (BDbiosciences). Data were analyzed using the FlowJo® software.

### Spheroid formation assay

Spheroid formation assay was performed as previously described (Paris et al. [30]). Cells (7000 cells/0.5 ml) were plated in 24-well plates coated with 1.5% agarose in complete RPMI medium and concentrated in the center by circular agitation. After three days, spheroids were recovered for inclusion in an extracellular matrix of collagen (100 μl) (final concentration = 2 mg/ml in buffer (0.01 N acetic acid; neutralization buffer: 33 mM Hepes pH 7.4, 0.37% sodium bicarbonate, 0.03 N NaOH; 1× MEM) in 24 well-plates coated with 1.5% agarose. Spheroids were maintained in complete medium with or without Erastin or IKE (1 µM) and images of the spheroids were captured over several days (0–7 days) using an Axio Vert.A1 inverted microscope (Carl Zeiss) at ×5 magnification. The invasion capacity was evaluated by determining the ratio between the maximum and initial area of the spheroid using ImageJ (Fiji 1.0).

### Wound-healing migration assay

Briefly, SKmel28 cells were grown to confluency in two-well silicone inserts (Ibidi^®^, Gräfelfing, Germany) and placed in 12-well tissue culture dishes. The cell culture insert was removed after one day. Afterwards, the plates were washed with PBS and incubated at 37 °C in fresh RPMI-1640 medium (Gibco BRL, Invitrogen, Paisley, UK) supplemented with 10% FBS (Eurobio Scientific, Evry, France) and 1% penicillin–streptomycin (Gibco, Invitrogen) in the presence of a vehicle (DMSO) or the indicated concentrations of Erastin or IKE. The wound was photographed at ×5 magnification using an Axio Vert.A1 inverted microscope (Carl Zeiss, Vision Aalen, Germany). Wound closure was determined by measuring the distance between the edges of the wound at 0 and 15 h using ImageJ software (Fiji, RRID:SCR_003070). The distance migrated by the cells was quantified as follows: D = (wound size at *t* = 0 h – wound size at *t* = 15 h).

### Apoptosis and necrosis assays

At the end of treatment, cells were stained by adding 25 µL of a dye mixture containing Hoechst33342 (5 µg/mL) propidium iodide (4 µg/mL), and YO-PRO^®^−1 (0.8 µM) directly into 100 µL of culture media and incubated at 37 °C for at least 45 min. Cells were imaged, analyzed, and counted using an ArrayScan™ VTI High-Content System (ThermoFisher Scientific, Courtaboeuf, France). Apoptotic (green) and necrotic (red) cells were expressed relative to the number of Hoechst-positive cells (blue).

### Metabolic flux assays

Adherent cells were seeded at 2 × 10^4^ cells/well in normal growth media (cell line specific) in an Agilent Seahorse XF96 (RRID:SCR_019545) cell culture microplate. To achieve an even distribution of cells within the wells, the plates were rocked at 25 °C for 20–40 min. For each staining group, one extra well on the outer perimeter of the plate was seeded to calibrate the image-acquisition parameters. The plate was then incubated at 37 °C overnight to allow cells to adhere. The following day, growth media were exchanged with Seahorse Phenol Red-free DMEM and either basal OCR was measured (for wells that were to be imaged with mitochondrial dyes, see Imaging section below) or an XF Cell Mito stress test (Agilent) was performed according to the manufacturer’s instructions. In both cases, the last injection port was used for cell stain/dye injection.

### Lipid Droplets analysis by microscopy

Briefly, the cells were rinsed in PBS and incubated with LipidSpot™-610 (1X in PBS) for 10 min at room temperature. Cells were washed twice with PBS and stained with WGA staining solution (5 μg/mL) for 10 min at 37° C. Cells were washed twice and incubated 5 min with DAPI staining solution before microscopy.

### RNA extraction and RT-qPCR expression

RNA extraction and RT-qPCR were performed as previously described [29]. The sequences of the primers used for RT-qPCR are in Supplementary Table 1 (EV2).

### Western blotting

Harvested cells were solubilized as described previously. Protein samples were denatured at 95 °C, resolved by SDS-PAGE, and transferred onto Hybond™-C Extra nitrocellulose membranes (Amersham Biosciences, Bucks, UK). The membranes were probed with the appropriate antibodies and the signals were detected using a Fujifilm LAS-3000 Imager (Fuji Photo Film, Tokyo, Japan). The primary antibody information is available in Supplementary Table 1 (EV3). Horseradish-peroxidase-conjugated secondary antibodies were purchased from Jackson ImmunoResearch (Suffolk, UK) and were used at a dilution of 1:10,000.

### RNA-seq

Total RNA was extracted from BRAFi-sensitive or -resistant SK28, Mel501, and M229 cells after knockout of AhR or not using the NucleoSpin RNA kit (Macherey Nagel, Düren, Germany). A complementary DNA library was prepared and sequenced according to the Illumina standard protocol by Beijing Novel Bioinformatics Co. Ltd (https://en.novogene.com/). RNAseq was performed in collaboration with Novogene (Beijing, China). Libraries were generated from 500 ng of total RNA using a Truseq Stranded mRNA Kit (Illumina). The concentration of the library was first determined using a Qubit2.0 fluorimter and then diluted to 1 ng/ul. The size of the insert was checked using an Agilent bioanalyzer and further quantified using qPCR (library concentration >2 nM). An aliquot (0.5 nM) of the pool was loaded onto a high-output flow cell and sequenced on a NovaSeq 6000 instrument (Illumina, RRID:SCR_016387) with 2 × 150 bp paired-end chemistry in two runs. Reads were aligned to the human genome release hg38 using HISAT2 V2.0.5 with default parameters. Quantification of the expressed genes was performed using CUFFDIFF v2.2.1 (RRID:SCR_001647). The quality of RNA-Seq count data was assessed using the Novogene standard protocol.

### Data mining

TCGA/SKCM RNAseq data were analyzed using the OncoLnc portal [http://www.oncolnc.org] (Anaya 2016). The raw data count matrix, composed of 454 samples (from the SKCM melanoma cohort), was downloaded from the OncoLnc portal for various transcriptional signatures. Expression heatmaps of differentially expressed genes between samples were obtained based on a log2 fold change using the ComplexHeatmap 2.0.0 (Gu, Eils, et Schlesner 2016, RRID:SCR_017270) package in R/Bioconductor. Cluster-specific gene rankings were obtained by comparing the samples to the rest. Cell density curves for the available melanoma cell lines were established using GraphPad PRISM 10.0^®^ (RRID:SCR_002798) to establish the IC50 values for the various treatments.

The raw data count matrices from the RNA seq data were obtained in GEO database (RRID:SCR_005012) for previous experiments on melanoma cell lines (Barretina et al. [33]) GSE36134 [https://www.ncbi.nlm.nih.gov/gds/?term=GSE36134] (sensitive or resistant to PLX470) (IC50 values for PLX4720 were obtained from Supplementary Table 7 of ref (Barretina et al. [33]), BRAFi- or BRAFi+MEKi-resistant cell lines GSE75299 [https://www.ncbi.nlm.nih.gov/gds/?term=GSE752099, (Song et al. 2017)] and GSE80829 [https://www.ncbi.nlm.nih.gov/gds/?term=GSE80829, (Tsoi et al. [19]) and GSE110054 [https://www.ncbi.nlm.nih.gov/geo/query/acc.cgi?acc=GSE110054, (Tsoi et al. [19]), BRAFi-treated melanoma patients (GSE65185 [https://www.ncbi.nlm.nih.gov/gds/?term=GSE65185, (Hugo et al. 2015)] and melanoma cell lines (proliferative or invasive) (GSE60664 [https://www.ncbi.nlm.nih.gov/gds/?term=GSE60664, (Verfaillie et al. 2015)].

Analysis of the RNAseq dataset from the Sanger/Massachusetts General Hospital Genomics of Drug Sensitivity in Cancer (GDSC) (Yang et al. 2013) was performed and recovered from the CellMinerCDB webtool (https://discover.nci.nih.gov/cellminercdb) (Reinhold et al. 2012). CellMinerCDB is an interactive web application that simplifies access to and exploration of cancer cell line pharmacogenomic data from different sources. This web tool allows the comparison of molecular and/or drug response patterns across sets of cell lines to search for possible associations. Pearson’s correlations with the reported p-values (not adjusted for multiple comparisons) between AhR expression (Supplemental Fig. 2B) and drug activity (297 compounds) were recovered for various cancer cell lines (*n* = 1080).

### Statistics

Data are presented as mean ± SD, unless otherwise specified, and differences were considered significant at *p* value < 0.05. Comparisons between groups normalized to a control were carried out using a two-tailed *t*-test, with the Holm-Sidak multiple comparisons test when more than two groups are compared to the same control condition. Overall survival was estimated using the Kaplan–Meier method. Univariate analysis using the Cox regression model was performed to estimate the hazard ratios (HR) and 95% confidence intervals (CI). All statistical analyses were performed using GraphPad (PRISM10.0^®^) (La Jolla, CA, USA).

### Patient-derived xenografts (PDXs)

The cutaneous melanoma PDX model is part of the Trace collection (https://gbiomed.kuleuven.be/english/research/50488876/54502087/Trace) and was established using leftover tissue from metastatic melanoma lesions derived from patients undergoing surgery as part of standard treatment at UZ Leuven. The patient was asked to sign a written informed consent, and procedures involving human samples were approved by the UZ Leuven/KU Leuven Medical Ethical Committee (S65645). The PDX model was used in accordance with the principles of the Declaration of Helsinki and with GDPR regulations. All the animal experiments were approved by the KU Leuven animal ethical committee under ECD P242/2024 and performed in accordance with the internal, national, and European guidelines of animal care and use. Mice were implanted with tumor pieces subcutaneously in the interscapular fat pad of NMRI mice and maintained in a semi-specific pathogen-free facility under standard housing conditions with continuous access to food and water. The health and welfare of the animals was supervised by a designated veterinarian. The KU Leuven animal facilities comply with all appropriate standards (cages, space per animal, temperature [22°C], light, humidity, food, and water), and all cages are enriched with materials that allow the animals to exert their natural behavior. Mice used in the study were maintained on a diurnal 12-h light/dark cycle. Fresh tumor tissue was collected in a transport medium (RPMI1640 medium supplemented with penicillin/streptomycin and amphotericin B). Tumor fragments were subsequently rinsed in phosphate-buffered saline supplemented with penicillin/streptomycin and amphotericin B and cut into small pieces of approximately 3 × 3 × 3 mm³. Tumor pieces were implanted in the interscapular fat pad of female SCID-beige mice (Taconic). After reaching generation 4 (F4), tumor fragments were implanted in the interscapular fat pad of female NMRI nude mice (8 weeks old, Taconic, RRID:IMSR_TAC:NMRI). Ketamine, medetomidine and buprenorphine were used for anesthesia.

### Pharmacologic treatment of mice

Mice with tumors reaching 100 mm³ were treated by daily oral gavage. IKE (Selleckchem) was dissolved in DMSO at a concentration of 30 mg/mL, respectively, aliquoted and stored at −80 °C. Each day a new aliquot was diluted 1:10 with phosphate-buffered saline and mice were treated with or without a dose of 30 mg/kg for IKE. Tumor volume was monitored with a caliper and calculated using the following formula: *V* = (*π*/6) × length × width × height. The endpoint of the experiment corresponded to when the tumor volume reached 2000 mm^3^ according to ethical statements.

## Supplementary information

Supplementary Table 1

Supplementary Table 2

Supplemented Figure 1

Supplemented Figure 2

Supplemented Figure 3

Supplemented Figure 4

Supplemented Figure 5

Supplemented Figure 6

Supplemental Figures legend

Full-length WB

## Data Availability

The datasets generated and/or analyzed during the current study are available from the corresponding author upon reasonable request. Full-length western blots are provided as supplementary material file.
