# Supplementary figures and images for: AhR-dependent ferroptosis as a therapeutic opportunity to counteract BRAFi-resistance in melanoma
Source: Cell Death Discov. 2026 Mar 23;12:303. doi: 10.1038/s41420-026-03057-3 (PMC13369207; doi:10.1038/s41420-026-03057-3)

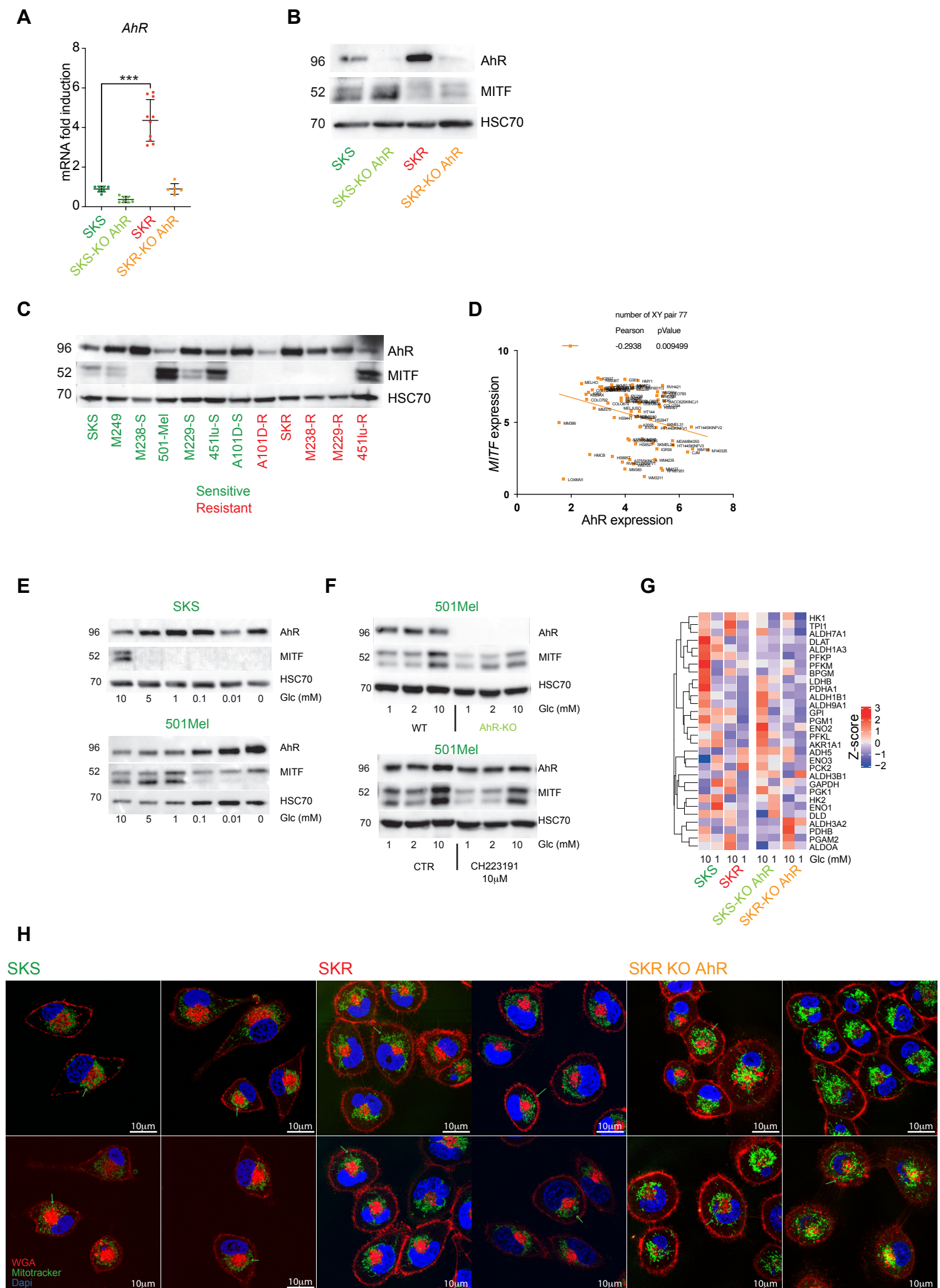

Appendix Figure S1

Supplement: Supplementary file 3 — Supplemented Figure 1 [file 41420_2026_3057_MOESM3_ESM.pdf]

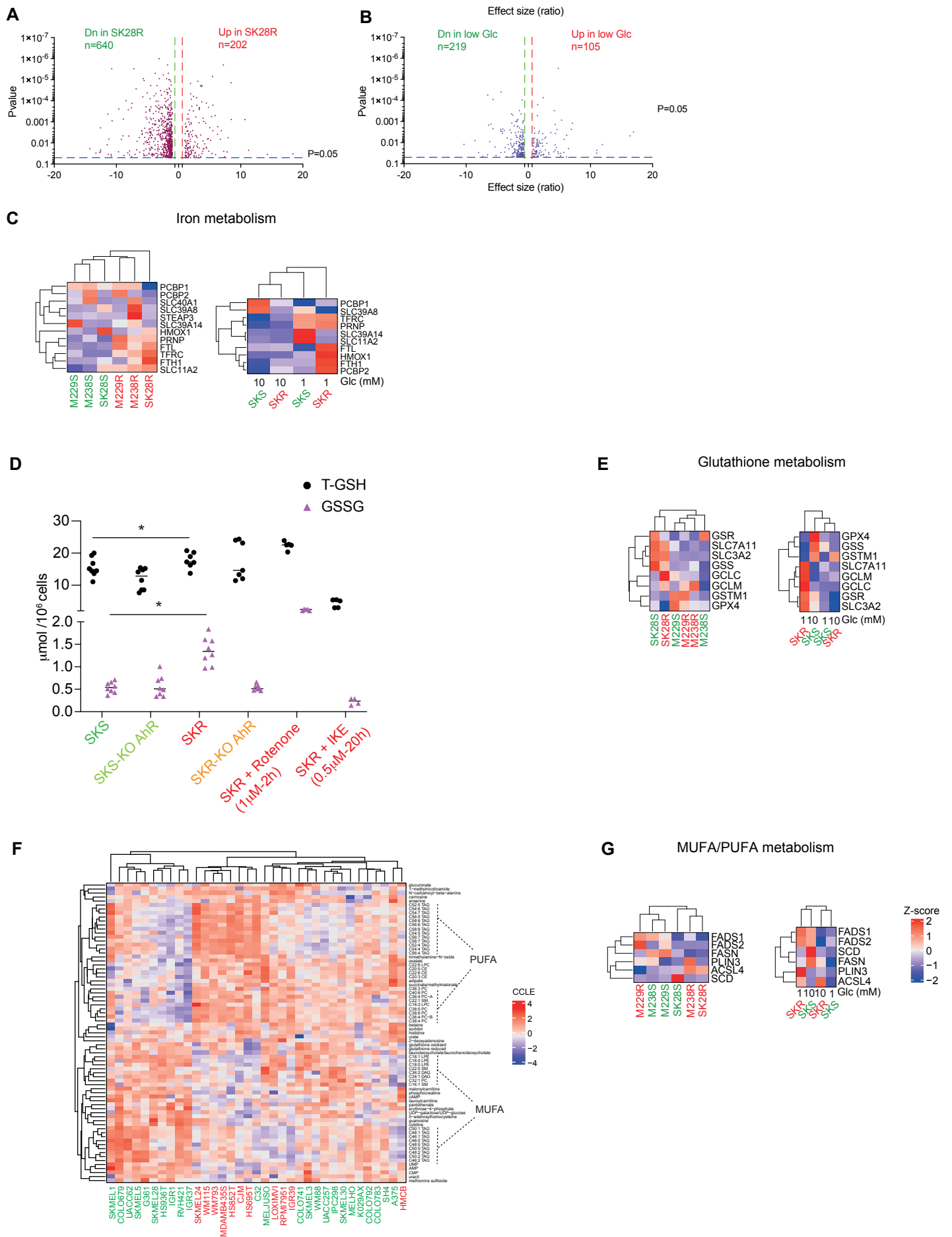

Supplement: Supplementary file 4 — Supplemented Figure 2 [file 41420_2026_3057_MOESM4_ESM.pdf]

**A**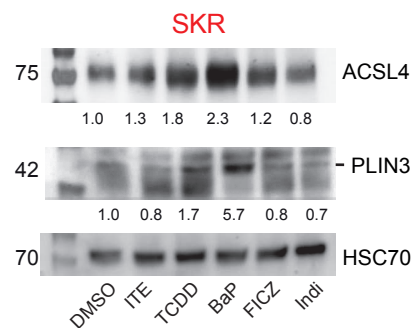**B**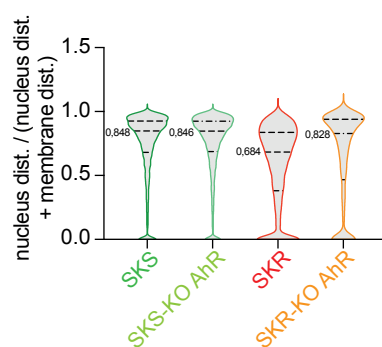**C**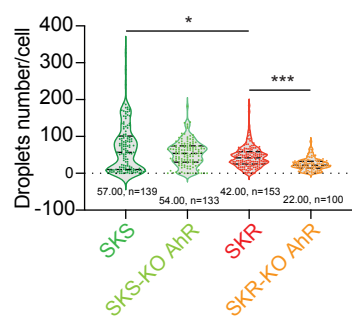**D**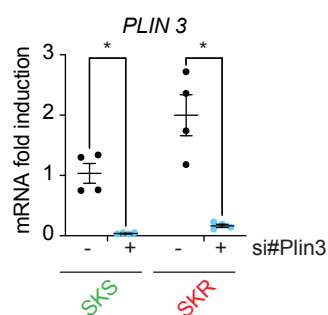**E**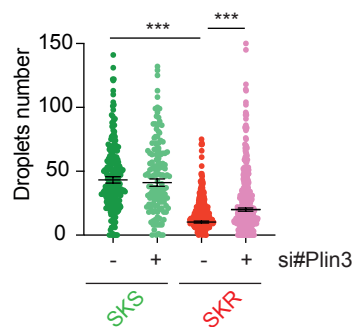**F**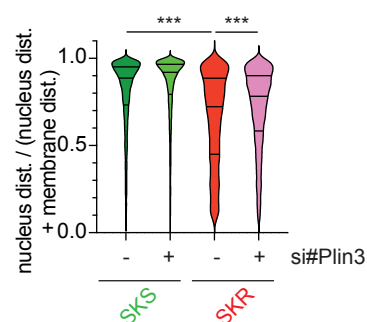**G**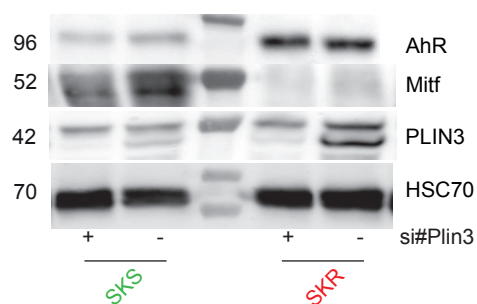

Supplement: Supplementary file 5 — Supplemented Figure 3 [file 41420_2026_3057_MOESM5_ESM.pdf]

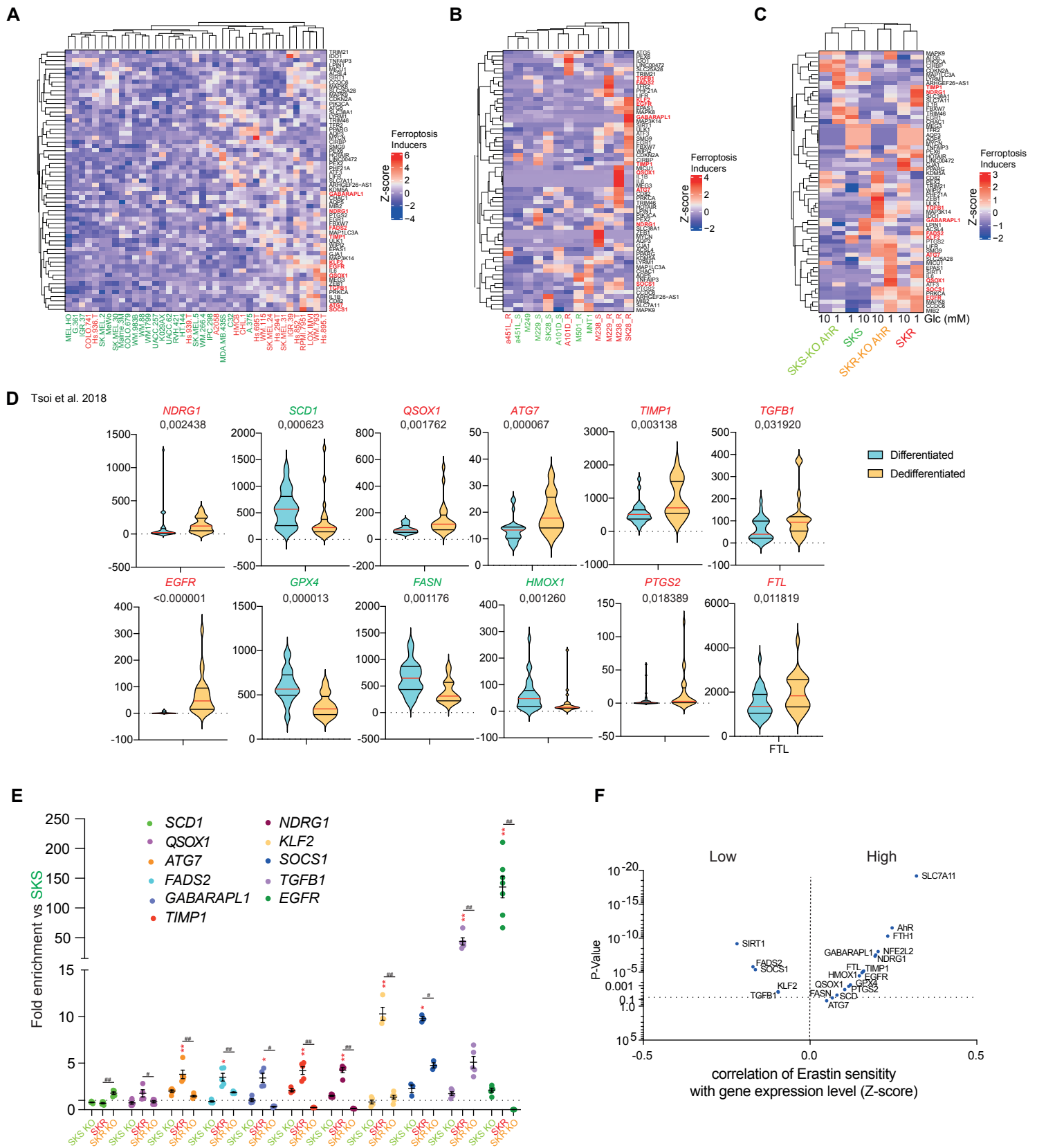

Supplement: Supplementary file 6 — Supplemented Figure 4 [file 41420_2026_3057_MOESM6_ESM.pdf]

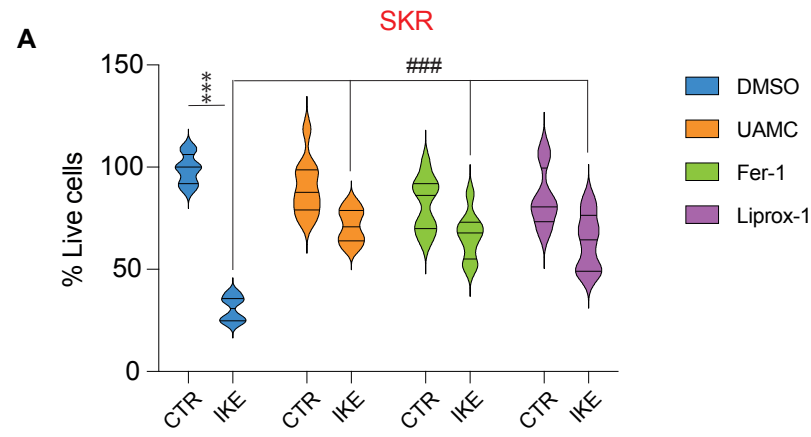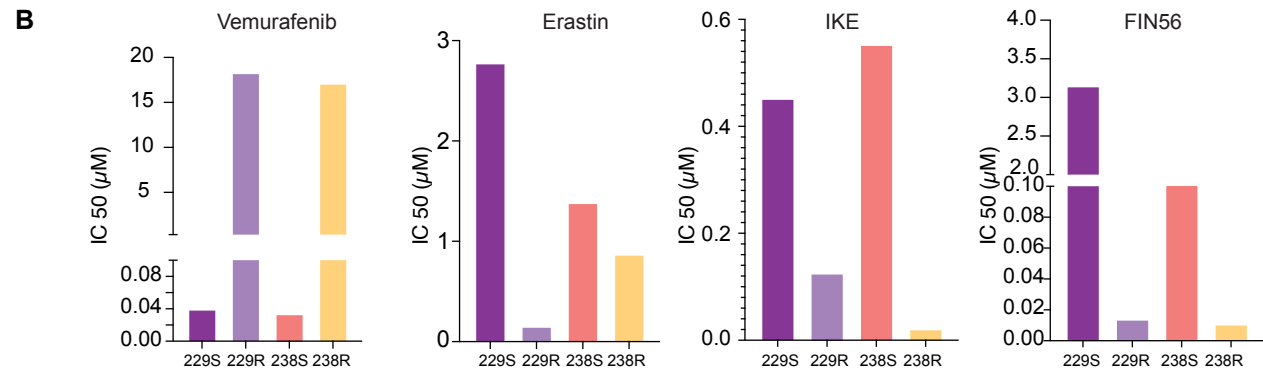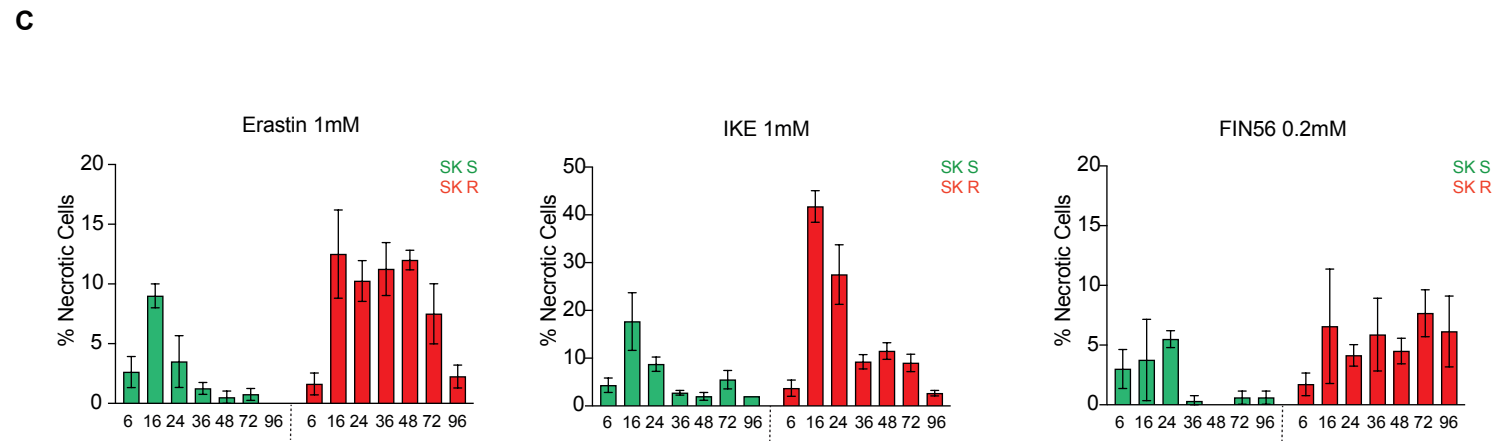

Supplement: Supplementary file 7 — Supplemented Figure 5 [file 41420_2026_3057_MOESM7_ESM.pdf]

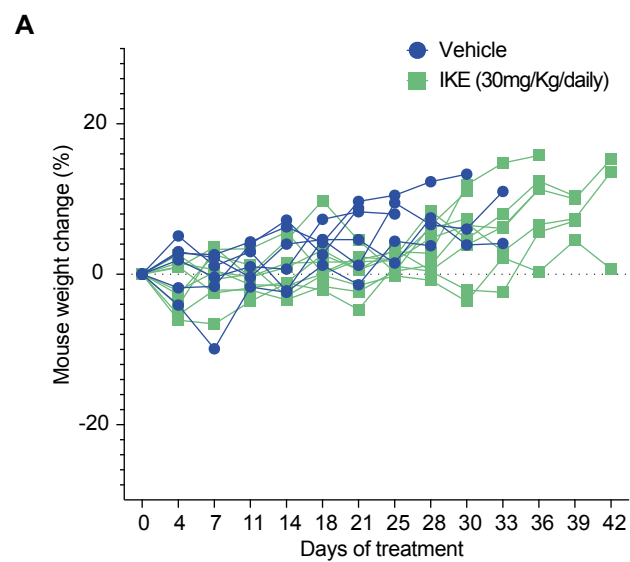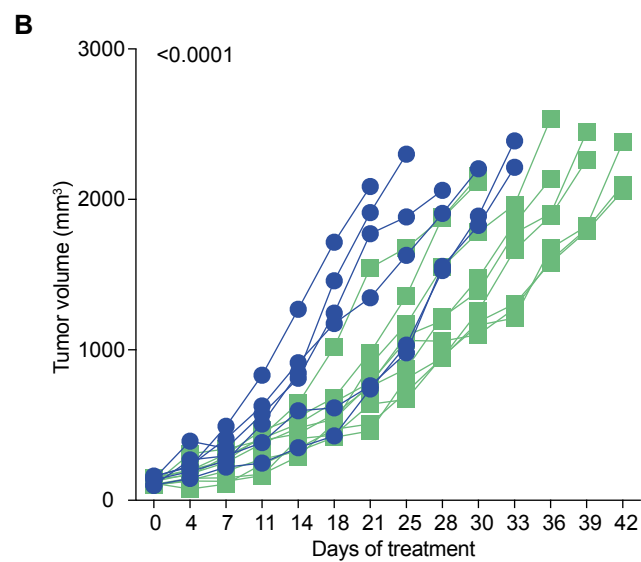

Supplement: Supplementary file 8 — Supplemented Figure 6 [file 41420_2026_3057_MOESM8_ESM.pdf]

Figure 1F

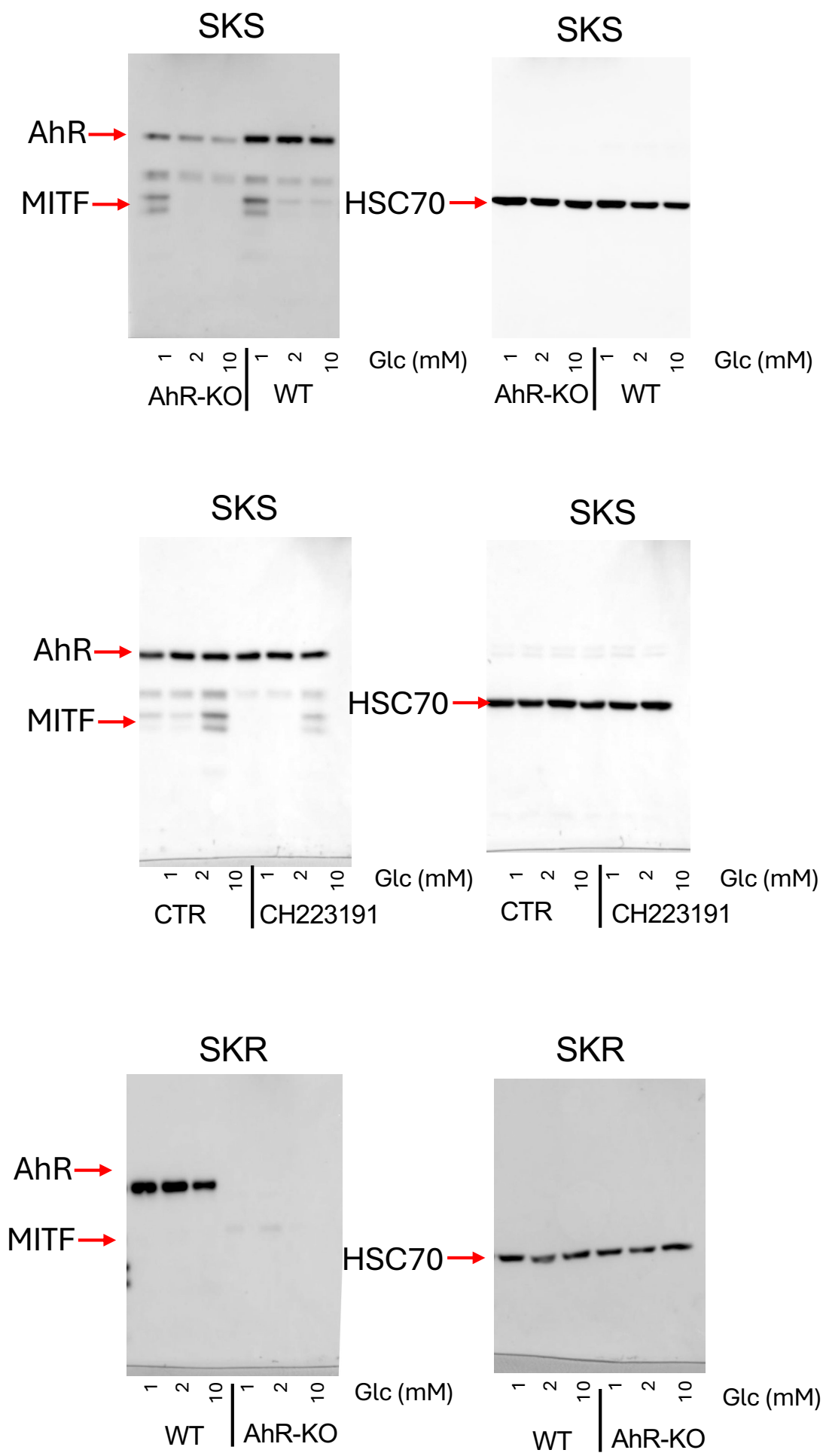

Figure 2B

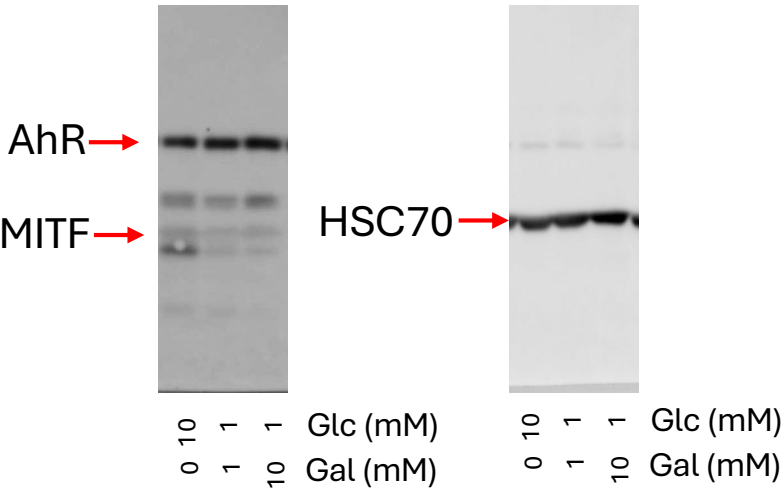

Figure 3H

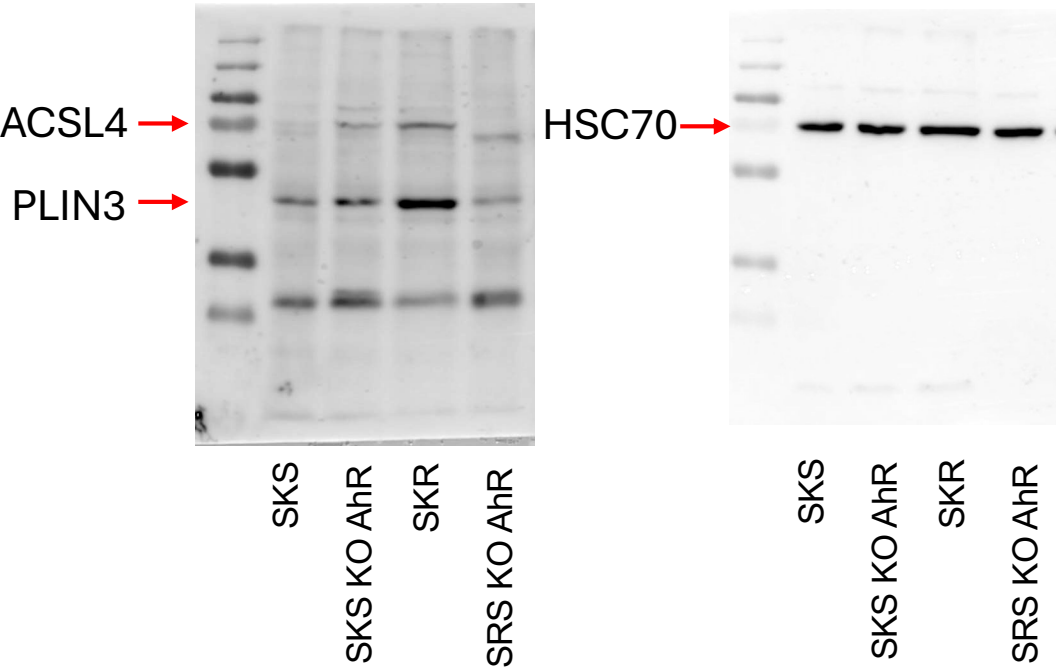

Figure S1C

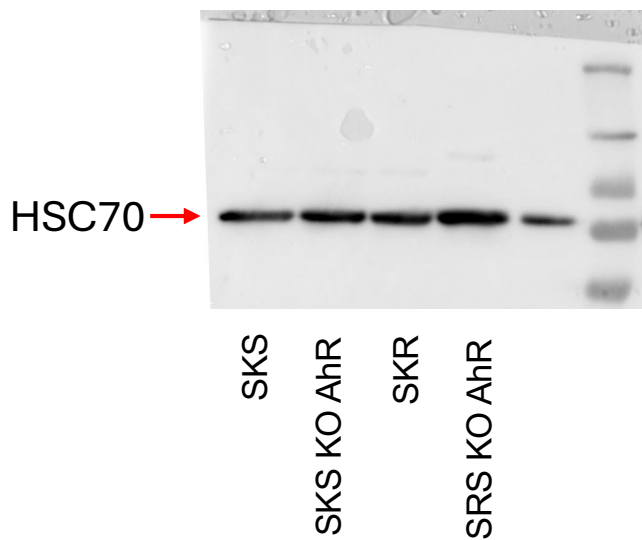

Figure S1C

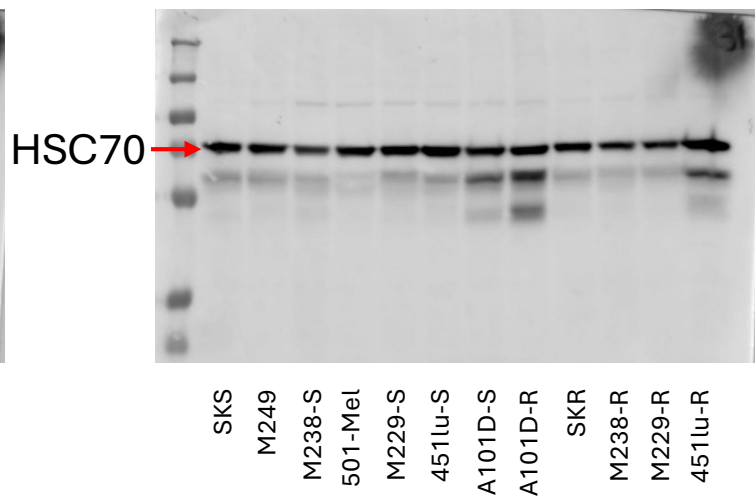

Figure S1E

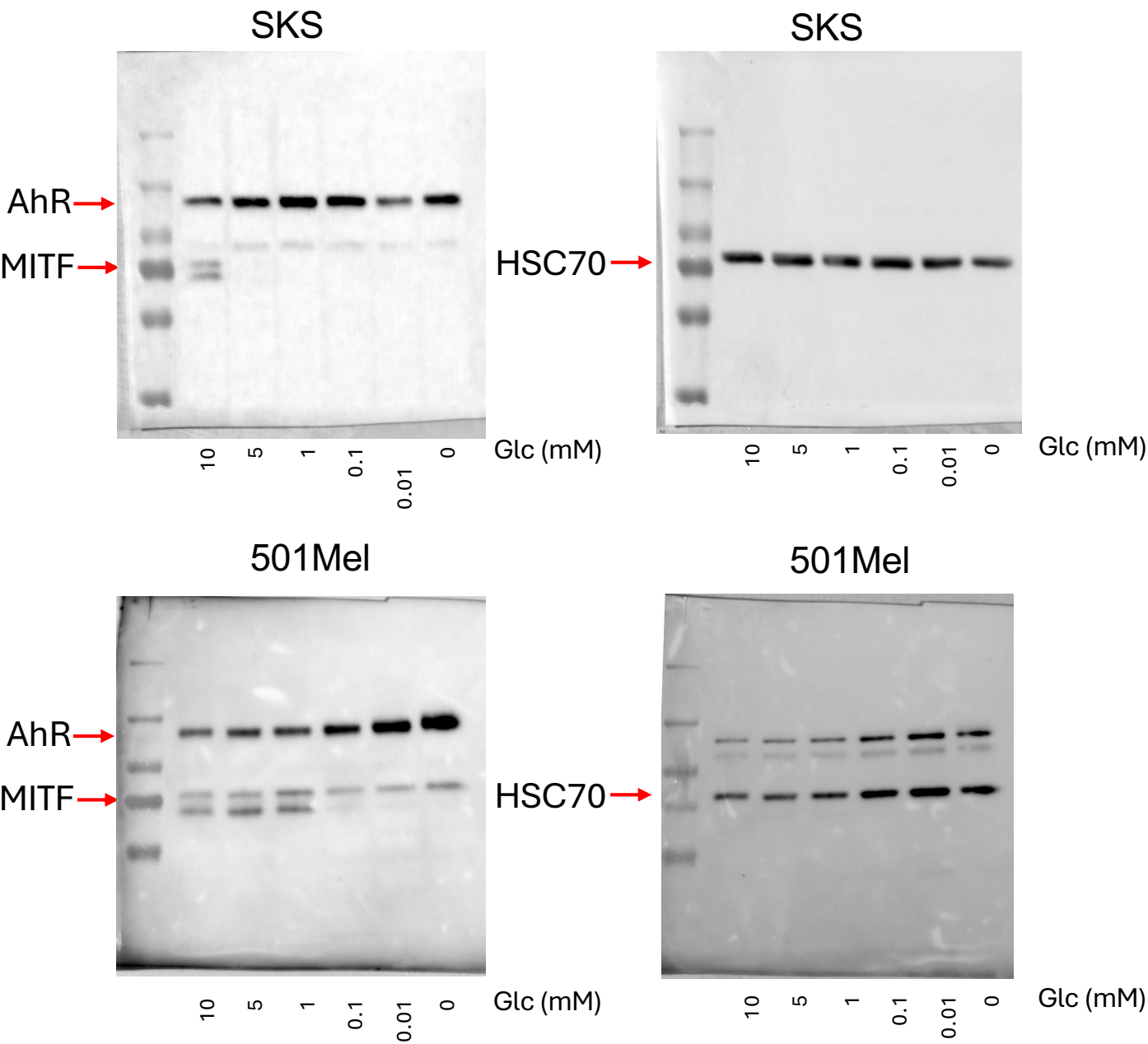

Figure S1F

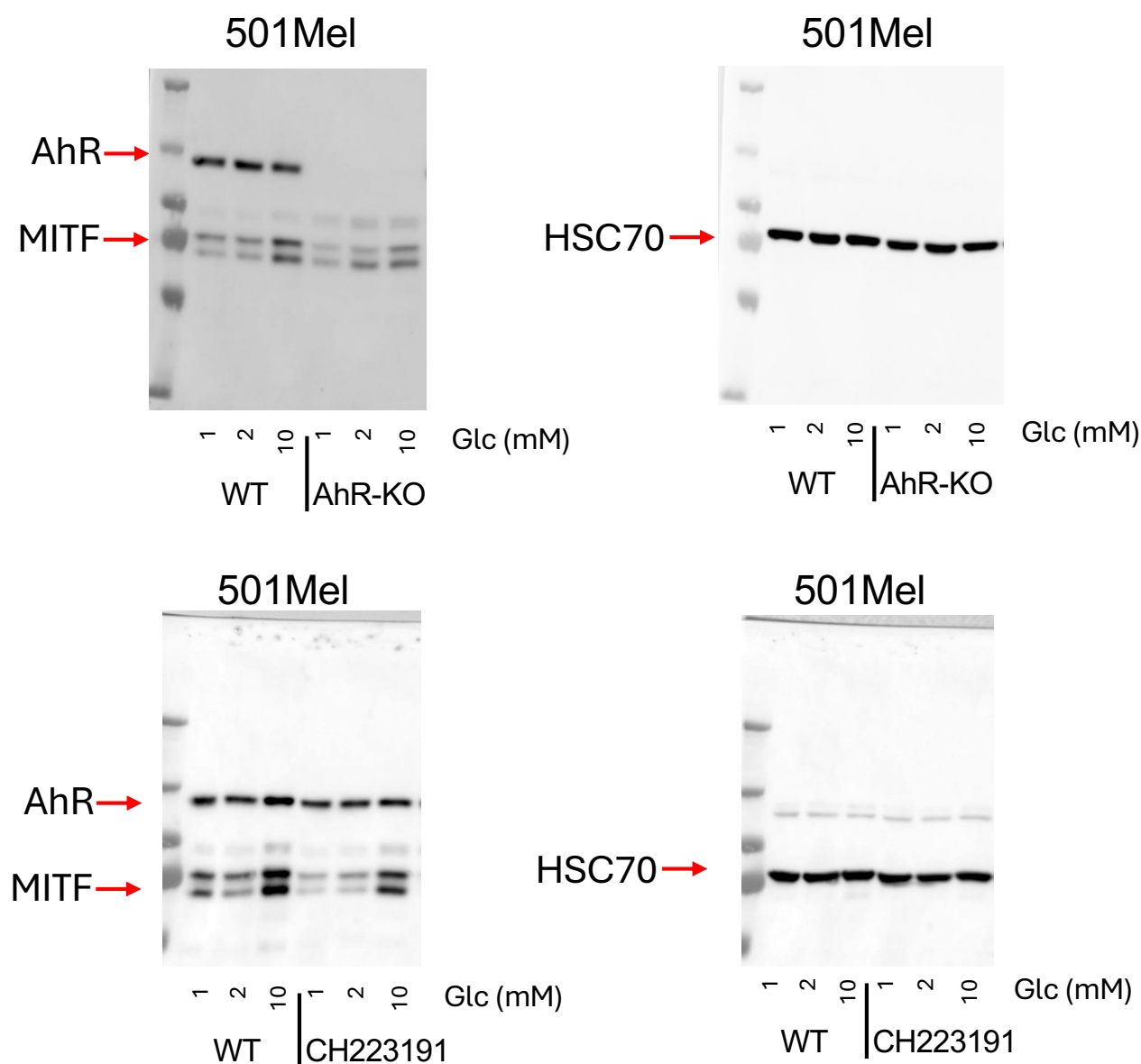

Figure S3C

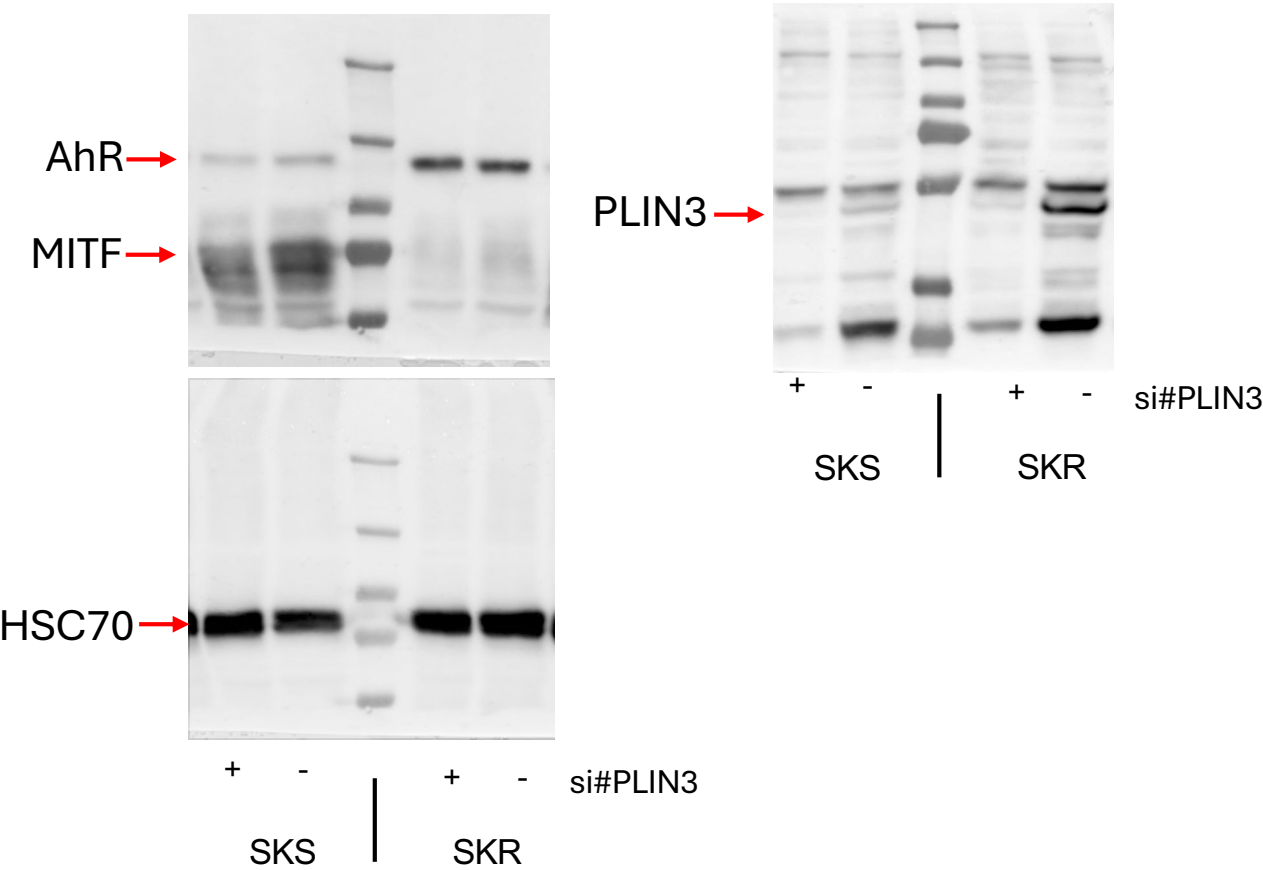

Supplement: Supplementary file 10 — Full-length WB [file 41420_2026_3057_MOESM10_ESM.pdf]
