# Supplementary material for: AhR-dependent ferroptosis as a therapeutic opportunity to counteract BRAFi-resistance in melanoma
Source: Cell Death Discov. 2026 Mar 23;12:303. doi: 10.1038/s41420-026-03057-3 (PMC13369207; doi:10.1038/s41420-026-03057-3)
Supplement: Supplementary file 9 — Supplemental Figures legend [file 41420_2026_3057_MOESM9_ESM.docx]

**Appendix Figure S1. Changes of expression of MITF and AhR transcription factors during energetic phenotype switch.**

**A**. *AhR* mRNA expression was measured by RT-qPCR in SKMel28 cells. Statistical analysis using unpaired *t*-tests was performed to compare the level of *AhR* expression to SKS, p<0.05 *; p<0.01 **; p<0.001 ***.

**B**. AhR protein levels in SKMel28 cells were analyzed by western blotting.

**C**. AhR and MITF protein levels were analyzed by western blotting in different BRAFi-sensitive (green) or -resistant (red) melanoma cell lines.

**D**. Analysis of correlation of mRNA expression between *AhR* and *MITF* from expression data in different melanoma cell lines (CCLE) using the Depmap webtool (<https://depmap.org/portal/interactive/>).

**E**. AhR and MITF protein levels were analyzed by western blotting in SKMel28 and 501Mel cells grown in the presence of increasing concentrations of glucose (0-10mM).

**F**. 501Mel cells invalidated or not for AhR (CRISPR-Cas9) or treated with its specific inhibitor (CH223191, 10 μM) were grown in increasing concentration of glucose (1, 2 and 10 mM). AhR and MITF protein levels were analyzed in these cells using western blotting.

**G**. Expression heatmap (results from RNAseq data) for various glycolytic genes in SKS and SKR, invalidated or not for AhR and grown in low (1 mM) or high glucose conditions (10 mM). Genes and clusters with similar expression profiles across the cohort were placed close to each other in the grid. The scale corresponds to Z scores.

**H**. Volcano plot combining the magnitude of the fold change (ratio of expression) for glycolytic genes between wildtype SKMel28 or invalidated for MITF (KO) and the p-values. Data were provided from supplemented tables by Dilshat et al [(Dilshat et al. 2021)](https://www.zotero.org/google-docs/?broken=Q1MqKt).

**Appendix Figure S2. Induction of iron, glutathione and lipid metabolism gene expression in BRAFi-resistant melanoma cells.**

**A**. Volcano plot combining the magnitude of the fold change (ratio of expression) of differentially expressed genes from RNAseq data between SKS (green, p>0.05 n=640) and SKR (red, p>0.05 n=202) and the p-values.

**B**. Volcano plot combining the magnitude of the fold change (ratio of expression) of differentially expressed genes from RNAseq data between SK grown in low glucose condition (1mM) and in high glucose condition (10mM) (down-regulated genes in green, p>0.05 n=219) (up-regulated genes in red, p>0.05 n=105) and the p-values.

**C-E-G**. Expression heatmap (results from RNAseq data) for genes involved in iron **(C)**, glutathione **(E)** and MUFA/PUFA lipid metabolism **(G)** in sensitive and resistant melanoma cell lines (SKMel28, M229 and M238) and in SKS and SKR, grown in low (1 mM) or high glucose condition (10 mM). The scale corresponds to the Z scores.

**D**. Measurement of total Glutathione (T-GSH) and oxidized glutathione (GSSG) levels in SKS, SKS KO, SKR and SKR KO at basal conditions. SKR reatment with rotenone or IKE was used as positive control for the accumulation of GSSG or for the reduction of glutathione (n=5-7; p<0.05).

**F**. Heatmap representing the levels of metabolites with differentially expressed levels between BRAFi-sensitive (green) or resistant (red) melanoma cell lines from the Cancer Cell Line Encyclopedia (CCLE).

**Appendix Figure S3. Lipid droplets distribution in SKS and SKR.**

**A**. ACSL4 and PLIN3 protein levels were analyzed by western blotting in SKR 24h after induction of cells with AhR ligands (ITE (5 μM), TCDD (10 nM), BaP (5 μM), FICZ (5 μM), and Indirubin (5 μM)).

**B**. Quantification of Lipid droplets (LD) after confocal microscopic analysis of lipid droplets (LD) in SKS and SKR cells invalidated or not for AhR after direct visualization by differential interference contrast microscopy (DIC). The graph represents the mean+/sem of the LD measured in >150 cells. p<0.05 *; p<0.001 ***.

**C**. The ratio between the distance to the nucleus and the sum of the distance to the nucleus and the distance to the plasma membrane was calculated for each LD (number of cells >150).

**D.** *PLIN3* mRNA expression and protein levels were measured using RT-qPCR and western blotting in SKMel28 cells 48h after transfection with and without specific siRNA.

**E**. Quantification of Lipid droplets (LD) after confocal microscopic analysis of lipid droplets (LD) in SKS and SKR 48h after transfection or not with *PLIN3* siRNA.

**F**. Sub-cellular distribution of lipid droplets in SKMel28 cells 48h after transfection with or without *PLIN3* siRNA.

**Appendix Figure S4. Analysis of expression of ferroptosis inducers in BRAFi sensitive and resistant melanoma cell lines.**

**A-C**. Expression heatmap for the different drivers of ferroptosis (<http://www.zhounan.org/ferrdb/current/>) in BRAFi sensitive (green) or resistant (red) melanoma cells using RNA seq data **(A)** from the Cancer Cell Line Encyclopedia (CCLE) [(Barretina et al. 2012)](https://www.zotero.org/google-docs/?broken=whKJzM) **(B)** from different BRAFi-sensitive or resistant melanoma cell lines and **(C)** from SKS and SKR, invalidated or not for AhR and grown in low (1 mM) or high glucose condition (10 mM).

**D**. Graph representing the variations in gene expression for different drivers of ferroptosis (<http://www.zhounan.org/ferrdb/current/>) in differentiated vs dedifferentiated melanoma cell lines from the Graeber data sets [(Tsoi et al. 2018)](https://www.zotero.org/google-docs/?broken=axm09Y).

**E**. Graph representing the variations in the expression of genes for different drivers of ferroptosis (<http://www.zhounan.org/ferrdb/current/>) in SKS KO AhR, SKR, SKR KO AhR compared to SKS (mean+/sem; n=4-6; p<0.05 *^, #^; p<0.01 **^, ##^; p<0.001 ***^, ###^).

**F.** Volcano plots showed a significant correlation (p < 0.001) between erastin sensitivity and the expression of ferroptosis markers and drivers’ genes.

**Appendix Figure S5. Measurement of IC50 of ferroptosis inducers.**

**A.** SKR cell density was established for 3 days after treatment with respective IC50 concentration of ferroptosis inducers IKE (SKR : 0,3μM) in the presence or not of specific ferroptosis inhibitor (UAMC 2 μM, Ferrostatin-1 2 μM and Liproxstatin-1 2μM). (mean+/sem; n=8; p<0.001 ***; p<0.001 ###).

**B.** Vemurafenib**,** Erastin, IKE and FIN56 sensitivity was established in M299S, M229R, M238S, and M238R melanoma cell lines by measuring cell density for four days after treatment (every 2 days), with an increasing concentration of ferroptosis inducers. The IC50 (nM) for each experiment was calculated using GraphPad (PRISM10.0).

**C.** SKS and SKR cells were treated with increasing concentrations of the inducers of ferroptosis, Erastin, IKE and FIN56. At different times after treatment, the cells were stained with Hoechst, propidium iodide, and YO-PRO^®^-1 to evaluate the percentage of necrotic cells relative to the number of Hoechst-positive cells by microscopy.

**Appendix Figure S6 Measurement of ferroptosis inducers.**

**A.** Vemurafenib**,** Erastin, IKE and FIN56 sensitivity was established in M299S, M229R, M238S and M238R melanoma cell lines by measuring cell density for four days after treatment (every 2 days), with an increasing concentration of ferroptosis inducers. The IC50 (nM) for each experiment was calculated using GraphPad (PRISM10.0).

**B.** SKS and SKR cells were treated with increasing concentrations of inducers of ferroptosis, Erastin, IKE and FIN56. At different times after the treatment, the cells were stained with Hoechst, propidium iodide, and YO-PRO^®^-1 to evaluate the percentage of necrotic cells relative to the number of Hoechst-positive cells by microscopy.

**Table Legends**

**Table 1 : Gene Set Enrichment analysis for Glycolysis signature in in CCLE (skin melanoma BRAFi: sensitive/resistant)** ^34^ **or in TSOI (skin melanoma differentiated/dedifferentiated)** ^19^ **datasets.**

**Table 2 : Supplemental material information.**
